# A chromosome-level reference genome of the hazelnut, *Corylus heterophylla* Fisch

**DOI:** 10.1093/gigascience/giab027

**Published:** 2021-04-19

**Authors:** Tiantian Zhao, Wenxu Ma, Zhen Yang, Lisong Liang, Xin Chen, Guixi Wang, Qinghua Ma, Lujun Wang

**Affiliations:** Research Institute of Forestry, Chinese Academy of Forestry/Key Laboratory of Tree Breeding and Cultivation of the State Forestry and Grassland Administration, No.1 Dongxiaofu, Xiangshan Road, Haidian District, Beijing 100091, China; National Hazelnut Industry Innovation Alliance/Hazelnut Engineering and Technical Research Center of the State Forestry and Grassland Administration, Xiangshan Road, Haidian District, Beijing 100091, China; Research Institute of Forestry, Chinese Academy of Forestry/Key Laboratory of Tree Breeding and Cultivation of the State Forestry and Grassland Administration, No.1 Dongxiaofu, Xiangshan Road, Haidian District, Beijing 100091, China; National Hazelnut Industry Innovation Alliance/Hazelnut Engineering and Technical Research Center of the State Forestry and Grassland Administration, Xiangshan Road, Haidian District, Beijing 100091, China; Research Institute of Forestry, Chinese Academy of Forestry/Key Laboratory of Tree Breeding and Cultivation of the State Forestry and Grassland Administration, No.1 Dongxiaofu, Xiangshan Road, Haidian District, Beijing 100091, China; National Hazelnut Industry Innovation Alliance/Hazelnut Engineering and Technical Research Center of the State Forestry and Grassland Administration, Xiangshan Road, Haidian District, Beijing 100091, China; Research Institute of Forestry, Chinese Academy of Forestry/Key Laboratory of Tree Breeding and Cultivation of the State Forestry and Grassland Administration, No.1 Dongxiaofu, Xiangshan Road, Haidian District, Beijing 100091, China; National Hazelnut Industry Innovation Alliance/Hazelnut Engineering and Technical Research Center of the State Forestry and Grassland Administration, Xiangshan Road, Haidian District, Beijing 100091, China; National Hazelnut Industry Innovation Alliance/Hazelnut Engineering and Technical Research Center of the State Forestry and Grassland Administration, Xiangshan Road, Haidian District, Beijing 100091, China; Shandong Institute of Pomology, Shandong Academy of Agricultural Sciences, No. 66 Longtan Road, Taishan District, Taian 271000, China; Research Institute of Forestry, Chinese Academy of Forestry/Key Laboratory of Tree Breeding and Cultivation of the State Forestry and Grassland Administration, No.1 Dongxiaofu, Xiangshan Road, Haidian District, Beijing 100091, China; National Hazelnut Industry Innovation Alliance/Hazelnut Engineering and Technical Research Center of the State Forestry and Grassland Administration, Xiangshan Road, Haidian District, Beijing 100091, China; Research Institute of Forestry, Chinese Academy of Forestry/Key Laboratory of Tree Breeding and Cultivation of the State Forestry and Grassland Administration, No.1 Dongxiaofu, Xiangshan Road, Haidian District, Beijing 100091, China; National Hazelnut Industry Innovation Alliance/Hazelnut Engineering and Technical Research Center of the State Forestry and Grassland Administration, Xiangshan Road, Haidian District, Beijing 100091, China; National Hazelnut Industry Innovation Alliance/Hazelnut Engineering and Technical Research Center of the State Forestry and Grassland Administration, Xiangshan Road, Haidian District, Beijing 100091, China; Anhui Academy of Forestry, No. 820 Changjiangxi Road, Shushan District, Hefei 230031, China

**Keywords:** *Corylus heterophylla* Fisch, Genome sequences, Nanopore, Hi-C

## Abstract

**Background:**

*Corylus heterophylla* Fisch. is a species of the Betulaceae family native to China. As an economically and ecologically important nut tree, *C. heterophylla* can survive in extremely low temperatures (–30 to –40 °C). To deepen our knowledge of the Betulaceae species and facilitate the use of *C. heterophylla* for breeding and its genetic improvement, we have sequenced the whole genome of *C. heterophylla*.

**Findings:**

Based on >64.99 Gb (∼175.30×) of Nanopore long reads, we assembled a 370.75-Mb *C. heterophylla* genome with contig N50 and scaffold N50 sizes of 2.07 and 31.33  Mb, respectively, accounting for 99.23% of the estimated genome size (373.61 Mb). Furthermore, 361.90 Mb contigs were anchored to 11 chromosomes using Hi-C link data, representing 97.61% of the assembled genome sequences. Transcriptomes representing 4 different tissues were sequenced to assist protein-coding gene prediction. A total of 27,591 protein-coding genes were identified, of which 92.02% (25,389) were functionally annotated. The phylogenetic analysis showed that *C. heterophylla* is close to *Ostrya japonica*, and they diverged from their common ancestor ∼52.79 million years ago.

**Conclusions:**

We generated a high-quality chromosome-level genome of *C. heterophylla*. This genome resource will promote research on the molecular mechanisms of how the hazelnut responds to environmental stresses and serves as an important resource for genome-assisted improvement in cold and drought resistance of the *Corylus* genus.

## Background

The *Corylus* genus, a member of the birch family Betulaceae that includes economically and ecologically important nut tree species, is widely distributed throughout temperate regions of the Northern Hemisphere [1]. As a valuable nut crop, hazelnut provides the predominant flavor in a variety of cakes, candies, chocolate spreads, and butters. There is a high content of unsaturated fatty acids and several essential vitamins in hazelnut oil.

The number of *Corylus* species recognized by taxonomists ranges from 7 to 25, depending on different morphological and molecular classifications [2, 3]. Among these, the European hazelnut, *Corylus avellana* L., is the most widely commercially cultivated species, with >400 cultivars having been described [4]. Commercial cultivation of *C. avellana* is limited to regions with climates moderated by large bodies of water that have cool summers and mild, humid winters, such as the slopes on the Black Sea of Turkey or the Willamette Valley of Oregon [5, 6]. Inadequate cold hardiness is a major factor limiting the expansion of commercial production into northern and inland areas. When *C. avellana* was introduced into China, twigs withered and died almost every winter owing to the cold, windy, and dry climate in northern China. In southern China, however, European hazelnut trees seemed to grow well but bore few nuts, and abortive kernels were observed frequently [7].

Eight species and 2 botanical varieties of *Corylus* are reported to be native to China [5]. The Asian hazel *Corylus heterophylla* (NCBI:txid80754) is one of the most important economic *Corylus* species. Among the 1.67 million ha of wild *Corylus* in China, *C. heterophylla* occupies 90% of the geographic area [8]. Wild *C. heterophylla* is mainly distributed in the mountains from northern to northeastern China. The geographical distribution range is 36.78–51.73 (°N) and 100.57–132.20 (°E), where the main climate type is temperate. Compared with *C. avellana, C. heterophylla* can be adapted to regions with low temperatures (–30 to –40 °C) and drought conditions. Therefore, the cold and drought resistance characteristics of *C. heterophylla* can be used as parent materials for cross-breeding with other hazel species.

In the present study, to better understand the molecular mechanism of how hazelnuts respond to environmental stress, we assembled a high-quality genome of *C. heterophylla* using a combination of the Oxford Nanopore high-throughput sequencing technology and the high-throughput chromosome conformation capture (Hi-C) technique. Long reads were *de novo* assembled into 1,328 polished contigs with a total size of 370.75 Mb and contig N50 and scaffold N50 values of 2.07 and 31.33 Mb, respectively, which is in line with genome sizes estimated using flow cytometry and *k*-mer analysis. A total of 361.90 Mb contigs were anchored into 11 chromosomes, representing 97.61% of the assembled genome. Our results provide a high-quality, chromosome-level genome assembly of *C. heterophylla*, which will support breeding programs leading to genetic improvement of hazelnuts. Furthermore, it will facilitate understanding of the special position of *Corylus* and Betulaceae in plant evolution.

## Data Description

### Sample collection

**Figure 1:**
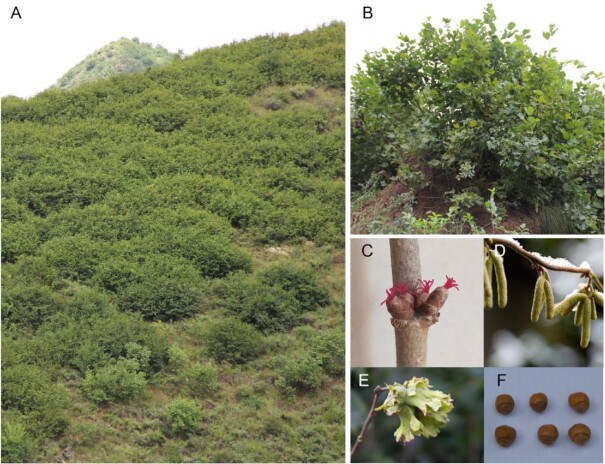
Morphological characteristics of the Asian hazelnut variety, *C. heterophylla*. Mature plants in (A) and (B), female inflorescence (C), staminate inflorescence (D), fruit with husk (E), and nuts (F) are shown.

Fresh and healthy leaves were collected from a single wild *C. heterophylla* tree in Yanqing, Beijing, China (40.54 N; 116.06 E; Fig. 1). The fresh leaf tissue was flash-frozen in liquid nitrogen for 30 min and then stored at –80 °C. DNA was extracted from leaf tissues following a previously published protocol [9]. Different tissues, including root, stem, staminate inflorescence, and leaf, were sampled and flash-frozen in liquid nitrogen for total RNA sequencing. Total RNA was extracted using the modified CTAB method [10].

### Library preparation and whole-genome sequencing

Genomic DNA for library construction was isolated from leaf tissues using the DNeasy Plant Mini Kit (Qiagen, Beijing, China) according to the manufacturer's instructions. DNA concentrations and quality were measured using a NanoDrop 2000 (Thermo Fisher, Waltham, MA, USA) and Qubit Fluorometer (Thermo Fisher, Waltham, MA, USA), respectively. The genomic DNA was sheared to ∼500-bp fragments using an S2 Focused-Ultrasonicator (Covaris Inc., Woburn, MA, USA). Paired-end (PE) libraries were prepared using the TruSeq DNA PCR-Free Library Preparation Kit (Illumina, San Diego, CA, USA) according to the Illumina standard protocol. After quality control by an Agilent 2100 Bioanalyzer and qPCR, all PCR-free libraries were sequenced on an Illumina HiSeq X Ten system (Illumina, San Diego, CA, USA) (Illumina HiSeq X Ten, RRID:SCR_016385) with a 350-bp PE sequencing strategy according to the manufacturer's instructions. A total of 38.02 Gb (∼102.55-fold coverage) clean reads were generated for the genome survey and Nanopore genome polishing (Supplementary Table S1a).

### Estimation of genome size and heterozygosity analysis

Before genome assembly, we estimated the *C. heterophylla* genome's size using Jellyfish (Jellyfish, RRID:SCR_005491) [11] with an optimal *k*-mer size. A total of 38.02 Gb short reads (∼102.55×) were processed by Jellyfish to assess their *k*-mer distribution (*k*-mer value = 19). Theoretically, the *k*-mer frequency follows a Poisson distribution. We selected *k* = 19 for the genome size estimation in this study. Genome sizes were calculated from the following equation: Genome size = 19-mer number/19-mer depth, where 19-mer number is the total counts of each unique 19-mer and 19-mer depth is the highest frequency that occurred (Supplementary Fig. S1). The estimated genome size of *C. heterophylla* is 373.61 Mb.

### Nanopore, RNA, and Hi-C sequencing

Genomic DNA was extracted and sequenced following the instructions of the Ligation Sequencing Kit (Oxford Nanopore Technologies, Oxford, UK). DNA quality was assessed by agarose gel electrophoresis and NanoDrop 2000c spectrophotometry, followed by Thermo Fisher Scientific Qubit fluorometry. After quality control, genomic DNA was size-selected using a Blue Pippin BLF7510 cassette (Sage Science, Beverly, MA, USA). Libraries (fragments >20 kb) were prepared using the standard Ligation Sequencing kit (SQK-LSK109; Oxford Nanopore Technologies, Oxford, UK) and sequenced on the GridION X5 platform (Oxford Nanopore Technologies, Oxford, UK) with FLOMIN106 (R9.4) flow cells. Raw ONT reads (fastq) were extracted from base-called FAST5 files using poretools [12]. Then, the short reads (<5 kb) and reads having low-quality bases and adapter sequences were removed. A total of 64.99 Gb (∼175.30-fold coverage) Nanopore long reads with an N50 length of 27.17 kb were produced for genome assembly (Supplementary Fig. S2, Supplementary Tables S1b and S1c).

Different tissues, including leaf, stem, root, and staminate inflorescence, were harvested and flash-frozen in liquid nitrogen for total RNA sequencing. Each sample was subjected to poly(A) purification using oligo-dT beads (Thermo Fisher, Waltham, MA, USA) followed by ribosomal RNA (rRNA) removal using the Ribo-Zero rRNA Removal Kit (Illumina, San Diego, CA, USA). The RNA quality was measured by 2100 RNA Nano 6000 Assay Kit (Agilent Technologies, Santa Clara, CA, USA) and pooling together. The resulting RNA sample was used for complementary DNA library construction using the NEBNext Ultra RNA Library Prep Kit for Illumina (NEB, Ipswich, MA, USA). The quantified libraries were then prepared for sequencing on the Illumina HiSeq X Ten system, producing 9.66 Gb PE reads (Supplementary Table S1d).

Hi-C experiments were performed as described with some modifications [13, 14]. Briefly, 2 g of freshly harvested leaves were cut into 2- to 3-mm pieces and infiltrated in 2% formaldehyde before cross-linking was stopped by adding glycine. The tissue was ground to powder and suspended in nuclei isolation buffer to obtain a nuclei suspension. The procedure for the Hi-C experiment, including chromatin digestion, labeling of DNA ends, DNA ligation, purification, and fragmentation, was performed as described previously [15]. The cross-linked DNA was digested with HindIII as previously described and marked by incubating with Klenow enzyme and biotin-14-dCTP overnight at 37°C [15]. The 5′ overhang of the fragments was repaired and labeled using biotinylated nucleotides, followed by ligation with T4 DNA polymerase. After reversal of cross-linking, ligated DNA was purified and sheared to 300–700 bp fragments using an S2 Focused-Ultrasonicator (Covaris Inc., MA, USA). The linked DNA fragments were enriched with streptavidin beads and prepared for Illumina HiSeq X Ten sequencing, producing 231.31 Mb (totaling ∼69.11 Gb) Hi-C link data (Supplementary Table S1e).

### *De novo* genome assembly and pseudo-chromosome construction

After the self-error correction using the error correction model in Canu (Canu, RRID:SCR_015880) v1.5 [16], the Nanopore long reads were assembled into contigs using WTDBG2 (WTDBG, RRID:SCR_017225) v1.0 [17]. Two rounds of consensus correction were performed using Racon (Racon, RRID:SCR_017642) v1.32 [18] with corrected Nanopore long reads, and the resulting assembly was further polished using Pilon (Pilon, RRID:SCR_014731) [19] with 38.02 Gb Illumina short reads (Supplementary Table S1a). The assembled length of 1,291 contigs of *C. heterophylla* is 370.71 Mb, accounting for 99.22% of the estimated genome size (373.61 Mb). The contigs N50 and N90 were 2.11 Mb and 138.6 kb, respectively.

The pseudo-chromosomes were constructed using Hi-C link data. The clean Hi-C reads were mapped to the consensus contigs using BWA [20] (BWA, RRID:SCR_010910) v0.7.17, and only uniquely mapped read pairs were considered as high-quality read pairs in Hi-C analysis. The reads were removed if the mapped positions in the reference genome were farther than 500 bp from the nearest restriction enzyme site. The quality assessment and normalization were performed using HiC-Pro (HiC-Pro, RRID:SCR_017643) [21]. There were 109,306,012 uniquely mapped PE reads, of which 58.33% (63,755,940) uniquely mapped reads were considered valid interaction pairs for chromosome construction (Supplementary Table S2). The contigs were then clustered, ordered, and oriented into 11 pseudo-chromosomes using LACHESIS (LACHESIS, RRID:SCR_017644) [21]. Finally, we obtained a high-quality chromosome-level reference genome with a total size of 370.75 Mb. The contig N50 and scaffold N50 values were 2.07  and 31.33  Mb, respectively (Table 1). A total of 361.90 Mb contigs were anchored into 11 chromosomes, representing 97.61% of the assembled genome (Table 2).

**Table 1:** Statistics of assembly results of *C. heterophylla* genome

Feature	*C. heterophylla*
Genome size (bp)	370,750,808
Contig	
No.	1,328
Maximum length (bp)	9,680,353
N50 (bp)	2,068,510
L50	48
N90 (bp)	125,301
Scaffold	
No.	951
Maximum length (bp)	46,514,939
N50 (bp)	31,328,411
L50	5
N90 (bp)	21,561,575
GC content (%)	35.84
Genes	
No.	27,591
Length (bp)	123,431,253
Mean length (bp)	4,473.61
Exons	
No.	138,886
Length (bp)	33,679,425
Introns	
No.	138,885
Length (bp)	89,751,828
Pseudogenes	
No.	2,988
Length (bp)	7,166,319

Note: only sequences of length >1 kb are considered.

**Table 2:** Summary of 11 pseudo-chromosomes for *C. heterophylla*

Chromosome	Clustered sequences		Ordered sequences	
	No.	Length (bp)	No.	Length (bp)
LG01	114	49,577,893	56	46,509,439
LG02	113	48,019,691	49	44,425,769
LG03	67	37,395,073	33	36,016,943
LG04	95	38,562,170	53	36,392,613
LG05	85	34,656,877	37	31,324,811
LG06	76	31,263,564	31	28,814,739
LG07	103	29,494,057	36	25,003,895
LG08	45	23,716,498	23	22,749,571
LG09	41	23,427,462	17	22,292,654
LG10	41	23,093,417	25	22,249,747
LG11	53	22,694,573	28	21,558,875
Total (%)	833 (62.73)	361,901,275 (97.61)	388 (46.58)	337,339,056 (93.21)

### Genome quality assessment

**Figure 2:**
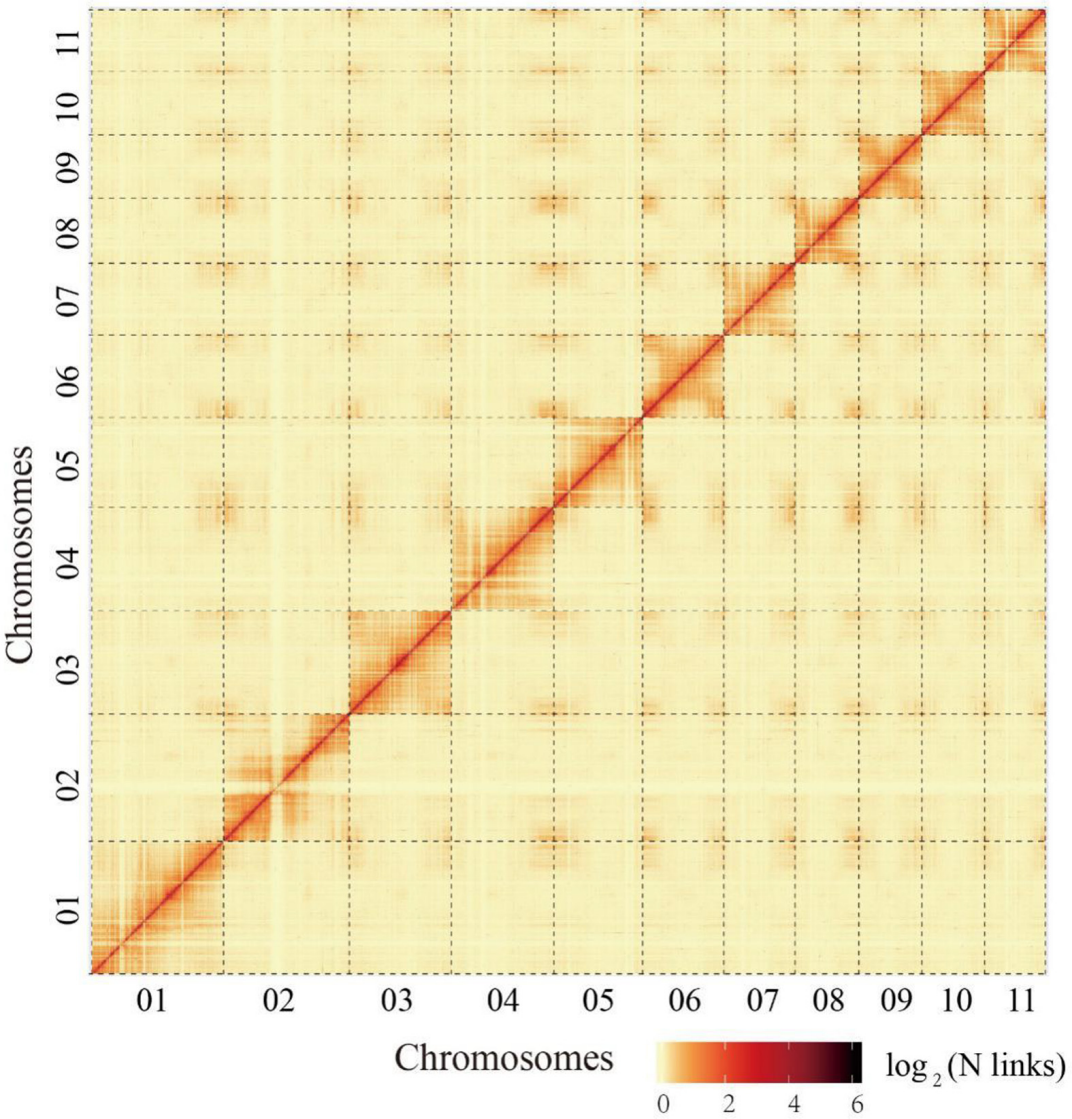
Interaction frequency distribution of Hi-C links among 11 chromosomes. Genome-wide Hi-C map of *C. heterophylla*. We scanned the genome by 500-kb nonoverlapping window as a bin and calculated valid interaction links of Hi-C data between any pair of bins. The log_2_ of link number was transformed. The color key of heat map ranging from light yellow to dark red represents the frequency of Hi-C interaction links from low to high (0–6).

Genome completeness was assessed using the plants dataset of the BUSCO (BUSCO, RRID:SCR_015008) database v1.22 [22], with e-value < 1e^−5^. The BUSCO database detected 93.47% and 1.18% of complete and partial gene models, respectively, in the *C. heterophylla* assembly results (Table 3). The core eukaryotic gene-mapping approach (CEGMA, RRID:SCR_015055) [23] provides a method to rapidly assess genome completeness because it comprises a set of highly conserved, single-copy genes, present in all eukaryotes, containing 458 core eukaryotic genes (CEGs). We identified CEGs using the CEGMA (CEGMA, RRID:SCR_015055) v2.3 pipeline [23] and found that 430 (93.89%) CEGs could be found in the assembly results (Supplementary Table S3a). The PE short libraries, including 103,392,992 paired reads, were remapped to the assembly genome with BWA-MEM (BWA, RRID:SCR_010910) [24] to assess the completeness of the assembly results. More than 98.47% of these reads could be accurately mapped into genome sequences (Supplementary Table S3b). Additionally, the heat map of the Hi-C interaction frequency was selected to visually assess the assembled accuracy of the *C. heterophylla* genome. The interaction heat map was displayed at 100-kb resolution. LG01–LG11 represent the 11 chromosomes of the *C. heterophylla* genome ordered by chromosome length. The horizontal and vertical coordinates represent the order of each “bin” on the corresponding chromosome. The signal intensities clearly divide the “bins” into 11 distinct groups (LG01–LG11), demonstrating the high quality of the chromosome assignment (Fig. 2). These observations suggest the high quality and completeness of this chromosome-level reference genome for *C. heterophylla*.

**Table 3:** Genome completeness assessment by BUSCO

BUSCO categories	No. (%)
Complete	1,346 (93.47)
Complete and single-copy	1,296 (90.00)
Complete and duplicated	50 (3.47)
Fragmented	17 (1.18)
Missing	77 (5.35)
Total groups searched	1,440 (100)

### Repetitive elements and protein-coding gene annotation

Repetitive elements in the *C. heterophylla* genome were identified using a combined strategy of *de novo* and homology-based approaches at the DNA and protein levels. Tandem repeats were annotated using TRF. A repeat library was constructed using MITE-Hunter (MITE-Hunter, RRID:SCR_020946) [25], LTR-FINDER (LTR_Finder, RRID:SCR_015247) v1.05 [26], RepeatScout (RepeatScout, RRID:SCR_014653) v1.0.5 [27], and PILER (PILER, RRID:SCR_017333) [28] for *de novo* repeat content annotation. The *de novo* repeat library was classified through PASTEClassifier (PASTEClassifier, RRID:SCR_017645) v1.0 package [29] with default parameters and then integrated with Repbase (RepeatMasker, RRID:SCR_012954) v19.06 [30] to build a new repeat library. Finally, RepeatMasker (RepeatMasker, RRID:SCR_012954) v4.0.6 [31] with parameters of “-nolow -no_is -norna -engine wublast” was selected to identify and classify the genomic repetitive elements of *C. heterophylla*. In total, 210.26 Mb of repetitive sequences were identified, accounting for 56.71% of *C. heterophylla* genome sequences (Table 4). The top 3 classes of repetitive elements were Class I/LARD, Class I/LTR/Gypsy, and Class I/LTR/Copia, occupying 20.51%, 11.14%, and 10.44% of assembled genome sequences, respectively (Table 4).

**Table 4:** Repetitive elements in the *C. heterophylla* genome

Class	No.	Length (bp)	Percent (%)
Class I	584,311	169,738,018	45.78
Class I/DIRS	18,638	7,059,337	1.9
Class I/LARD	303,288	76,033,830	20.51
Class I/LINE	60,182	18,890,786	5.1
Class I/LTR/Copia	101,158	38,719,023	10.44
Class I/LTR/Gypsy	83,300	41,302,761	11.14
Class I/LTR/Unknown	1,953	1,080,718	0.29
Class I/PLE	5,600	4,125,513	1.11
Class I/SINE	5,344	1,058,985	0.29
Class I/TRIM	3,828	1,023,113	0.28
Class I/Unknown	1,020	244,561	0.07
Class II	77,407	24,382,510	6.58
Class II/Crypton	455	109,226	0.03
Class II/Helitron	27,254	8,348,317	2.25
Class II/MITE	1,112	194,088	0.05
Class II/Maverick	754	165,986	0.04
Class II/TIR	44,403	15,342,483	4.14
Class II/Unknown	3,429	459,116	0.12
Potential host gene	46,369	9,994,181	2.7
SSR	1,135	265,113	0.07
Unknown	116,728	26,584,597	7.17
Total	825,950	210,255,221	56.71

DIRS: dictyostelium intermediate repeat sequence; LARD: large retrotransposon derivative; LINE: long interspersed nuclear element; LTR: long terminal repeat; MITE: miniature inverted-repeat transposable element; PLE: Penelope-like element; SINE: short interspersed nuclear element; SSR: simple sequence repeat; TIR: terminal inverted repeat; TRIM: terminal-repeat retrotransposons in miniature.

Gene annotation was performed using a combination of *ab initio* prediction, homology-based gene prediction, and transcript evidence from RNA-seq data. The *de novo* approach was implemented using Augustus (Augustus, RRID:SCR_008417) v3.2.3 [32], GeneID (Entrez Gene, RRID:SCR_002473) v1.4.4 [33], GlimmerHMM (GlimmerHMM, RRID:SCR_002654) v3.52 [34], GenScan (GENSCAN, RRID:SCR_012902) [35], and SNAP (SNAP, RRID:SCR_007936) [36]. For homology-based prediction, TBLASTN (TBLASTN, RRID:SCR_011822) v2.2.31 [37] was used to align predicted protein sequences of *Arabidopsis thaliana*,  *Betula pendula*,  *Juglans regia*, and *Ostrya chinensis* to the *C. heterophylla* genome with an e-value threshold of 1e^−5^. Then, GeMoMa (GeMoMa, RRID:SCR_017646) v1.3.1 [38] was used for homology-based gene prediction. The transcriptome data from pooled tissues of leaf, stem, root, and staminate inflorescence from *C. heterophylla* were assembled into unigenes using HISAT (HISAT, RRID:SCR_015530) v2.0.4 [39] and StringTie (StringTie, RRID:SCR_016323) v1.2.3 [40]. Then unigenes were used to predict gene structures using TransDecoder (TransDecoder, RRID:SCR_017647) v2.0 [41], GeneMarkS-T (GeneMarkS-T, RRID:SCR_017648) v5.1 [42], and PASA (PASA, RRID:SCR_014656) v2.0.2 [43]. Finally, the gene models obtained from the above 3 approaches were integrated into a consensus gene set using EVidenceModeler (EVidenceModeler, RRID:SCR_014659) v1.1.0 [44] with default parameters. PASA (PASA, RRID:SCR_014656) v2.0.2 [43] was then used to annotate the gene structures, including untranslated regions and alternative-splice sites (Supplementary Fig. S3, Supplementary Table S4a). A total of 27,591 non-redundant protein-coding genes were predicted for the *C. heterophylla* genome (Table 1). Gene models were annotated by homologous searching against several databases using BLASTP (BLASTP, RRID:SCR_001010) from the BLAST+ package [37] (e-value = 1e^−5^), including NR [45], KOG [46], TrEMBL (Universal Protein Resource, RRID:SCR_002380) [47], and KEGG (KEGG, RRID:SCR_012773) [48] databases. InterProScan (InterProScan, RRID:SCR_005829) v4.3 [49] was used to annotate the protein motifs and domains. The Blast2GO (Blast2GO, RRID:SCR_005828) [50, 51] pipeline was used to obtain GO terms annotation from the NCBI NR database. In total, 25,389 protein-coding genes (92.02%) were successfully assigned into corresponding functions (Supplementary Table S4b).

Genome-wide pseudogene identification was carried out for *C. heterophylla*. Only candidate pseudogenes containing frameshifts and/or premature stop codons in their coding regions were considered as reliable pseudogenes. *C. heterophylla* proteins were aligned to the reference genome using GenBlastA (GenBlastA, RRID:SCR_020951) v1.0.4 [52] to detect candidate homolog regions. Then, the candidate pseudogenes were identified using GeneWise (GeneWise, RRID:SCR_015054) v2.4.1 [53]. Finally, 2,988 pseudogenes were identified in *C. heterophylla* genome sequences (Table 1).

Different types of non-coding RNA in the *C. heterophylla* genome were identified and classified as family and subfamily. The tRNAscan-SE (tRNAscan-SE, RRID:SCR_010835) v1.23 [54] was applied to detect transfer RNAs (tRNAs). MicroRNAs (miRNAs) were identified by homolog searching miRBase (microRNA database (miRBase), RRID:SCR_003152) v21 [55] against the *C. heterophylla* genome with 1 mismatch. Then, secondary structures of the putative sequences were predicted by miRDeep2 (miRDeep, RRID:SCR_010829) [56]. Finally, putative miRNAs with hairpin structures were considered as reliable ones. Other types of non-coding RNA were detected using Infernal (Infernal, RRID:SCR_011809) [57] (e-value ≤ 0.01) based on the Rfam database (Rfam, RRID:SCR_007891) v12.0 [58]. In total, 92 miRNAs, 617 tRNAs, and 622 rRNAs were annotated in *C. heterophylla* genome sequences (Supplementary Table S4c).

### Gene family identification and phylogenetic tree construction

**Figure 3:**
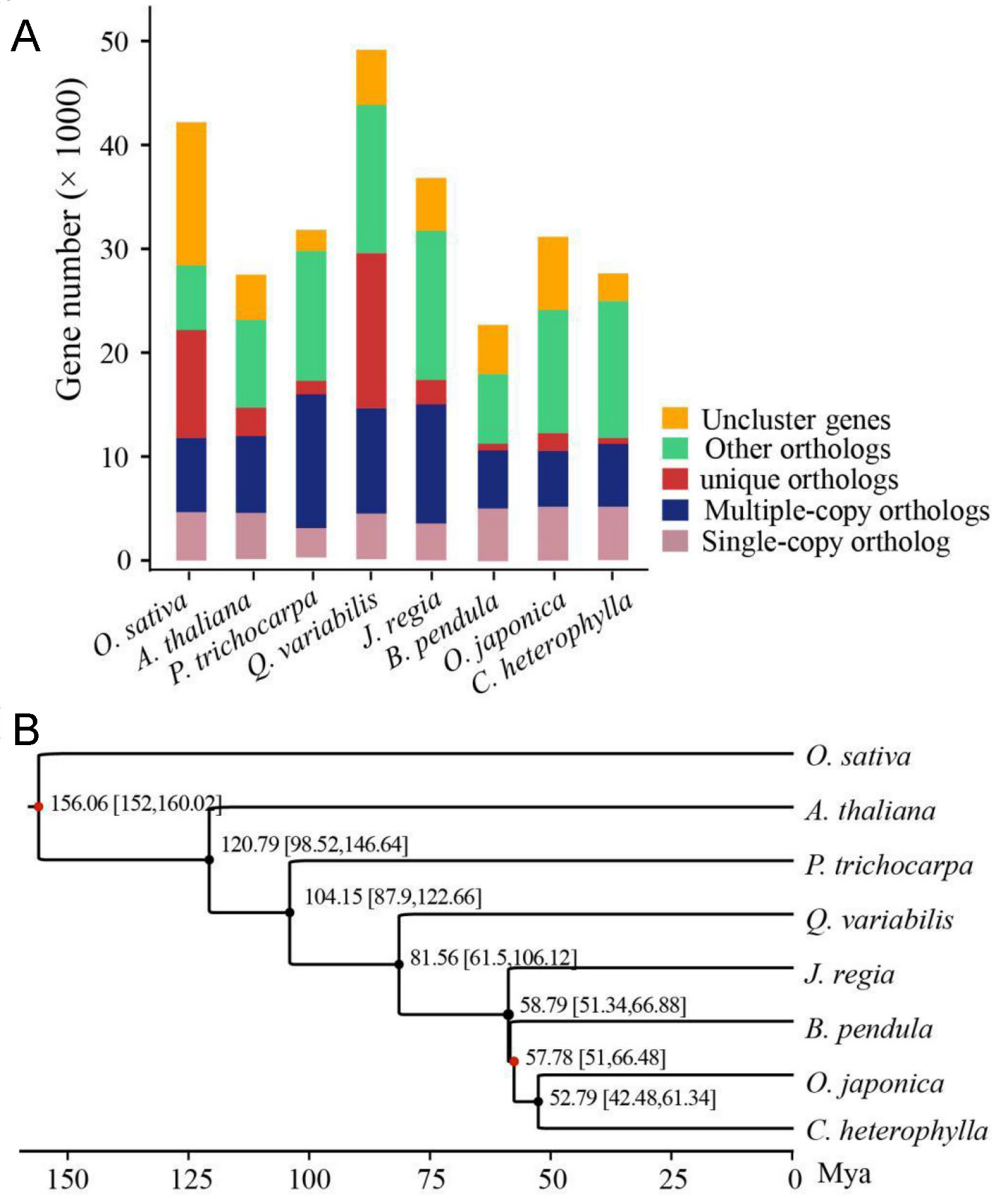
Genome evolution analysis of *C. heterophylla*. (A) Summary of gene family clustering of *C. heterophylla* and 7 related species. Single-copy orthologs: 1-copy genes in ortholog group. Multiple-copy orthologs: multiple genes in ortholog group. Unique orthologs: species-specific genes. Other orthologs: the rest of the clustered genes. Uncluster genes: number of genes out of cluster. (B) Phylogenetic relationship and divergence time estimation. The *O. sativa* was considered as outgroup in phylogenetic tree construction. The red dots indicate the fossil correction time of *O. sativa* vs *P. trichocarpa* (152–160 Mya) and crown nodes of family Betulaceae (51.2–66.7 Mya), respectively.

In the gene family and phylogenetic analysis, the protein-coding genes of *O. sativa, A. thaliana, Populus trichocarpa, Quercus variabilis, J. regia, B. pendula, Ostrya japonica*, and *C. heterophylla* were downloaded from Genbank or Ensembl databases. The longest transcripts were selected to represent the protein-coding genes. Protein sequence clustering was performed using OrthoMCL (OrthoMCL DB: Ortholog Groups of Protein Sequences, RRID:SCR_007839) v2.0 [59] with default parameters to identify the orthologous groups. The result showed that *C. heterophylla* has 16,811 orthologous groups, including 5,150 single-copy genes, 6,040 multiple-copy genes, and 582 specific genes. Notably, 222 species-specific families were identified for *C. heterophylla*, which might contribute to its unique features (Fig. 3A). To construct the phylogenetic analysis, 1,182 single-copy orthologs were identified from 1-copy families of selected species. The protein sequences of single-copy orthologs were aligned using MUSCLE (MUSCLE, RRID:SCR_011812) v3.8.31 [60], and low-quality alignment regions were removed using Gblocks (Gblocks, RRID:SCR_015945) v0.91b [61] with default parameters. A phylogenetic tree was constructed using the maximum-likelihood method with the JTT amino acid substitution model implemented in the PhyML (PhyML, RRID:SCR_014629) v3.3 package [62]. The divergence time was estimated using the MCMCtree program in the PAML (PAML, RRID:SCR_014932) v4.7b package [63]. An age of 51.2–66.7 million years ago (Mya) was used to calibrate the crown nodes of the family Betulaceae [64]. The monocot-dicot split time (152–160 Mya) obtained from the TimeTree database was also used to calibrate the time estimation [65]. The result showed that *C. heterophylla* is close to *O. japonica*, and they diverged from their common ancestor ∼52.79 Mya (Fig. 3B).

## Conclusion

To our knowledge, this is the first report of a chromosome-level genome assembly of *C. heterophylla* using the third-generation sequencing technologies of Nanopore and Hi-C. *C. heterophylla* has 210.26 Mb of repetitive sequences, accounting for 56.71% of genomic sequences. A total of 25,389 high-quality protein-coding genes were annotated by integrating evidence from *de novo* prediction, homologous protein prediction, and transcriptome data. Phylogenetic analysis showed that *Corylus* is closely related to *Ostrya*, and they diverged from their common ancestor ∼52.79 Mya. This work provides valuable chromosome-level genomic data for studying hazelnut traits. The genomic data should promote research on the molecular mechanisms of hazelnut responses to environmental stress and provides a valuable resource for genome-assisted improvements in *Corylus* breeding.

## Data Availability

The genome sequence data underlying this article are available in NCBI and can be accessesd with accession JADOBO000000000. Raw reads of Nanopore, whole-genome sequencing, Hi-C, and RNAseq, and genome assembly sequences of the *C. heterophylla* genome have been deposited at the Nucleotide Sequence Archive and GenBank in NCBI under BioProject PRJNA655406 and BioSample Accessions of SAMN15734705 and SAMN15734794. The SRA accessions are SRR12458330, SRR12458329, SRR12458328, and SRR12458327. Additional supporting data and materials, including annotations, RNA-seq data, and phylogenetic trees, are available in the GigaScience databse, GigaDB [66].

## Additional Files

Supplementary Figure S1: Genome survey analysis of *C. heterophylla* based on *k*-mer = 19.

Supplementary Figure S2: Fragment size distribution of Hi-C read pairs.

Supplementary Figure S3: Venn plot of predicted genes generated from *ab initio*, RNAseq, and homology methods.

Supplementary Table S1a: Summary of Illumina data for genome survey and genome polishing.

Supplementary Table S1b: Statistics of Nanopore long reads.

Supplementary Table S1c: Distribution of Nanopore long-read lengths.

Supplementary Table S1d: Summary of pooled transcriptome data used for gene prediction.

Supplementary Table S1e: Summary of Hi-C data for error correction and chromosome construction.

Supplementary Table S2: Valid interaction pairs of Hi-C sequencing data.

Supplementary Table S3a: Completeness analysis based on the CEG database.

Supplementary Table S3b: Genome completeness assessment based on Illumina sequencing reads.

Supplementary Table S4a: Summary of gene predictions resulting from different evidence.

Supplementary Table S4b: Gene function annotated by different databases.

Supplementary Table S4c: Non-coding RNA identification.

## Abbreviations

BLAST: Basic Local Alignment Search Tool; bp: base pairs; BUSCO: Benchmarking Universal Single-Copy Orthologs; BWA: Burrows-Wheeler Aligner; CEGMA: Core Eukaryotic Genes Mapping Approach; CTAB: hexadecyltrimethylammonium bromide; Gb: gigabase pairs; GeMoMa: Gene Model Mapper; GO: Gene Ontology; Hi-C: high-throughput chromosome conformation capture; HiSeq: high-throughput sequencing; HMM: hidden Markov model; kb: kilobase pairs; KEGG: Kyoto Encyclopedia of Genes and Genomes; KOG: EuKaryotic Orthologous Groups; LG: linkage group; LTR: long terminal repeat; Mb: megabase pairs; miRNA: microRNA; MITE: miniature inverted-repeat transposable element; MUSCLE: MUltiple Sequence Comparison by Log-Expectation; Mya: million years ago; NCBI: National Center for Biotechnology Information; NR: non-redundant; PAML: Phylogenetic Analysis of Maximum-Likelihood; PASA: Program to Assemble Spliced Alignments; PE: paired-end; PhyML: Phylogeny Maximum Likelihood; RNA-seq: RNA sequencing; rRNA: ribosomal RNA; SNAP: Semi-HMM-based Nucleic Acid Parser; TIR: terminal inverted repeat; TrEMBL: a database of translated proteins from European Bioinformatics Institute; TRF: Tandem Repeats Finder; tRNA: transfer RNA.

## Competing Interests

The authors declare that they have no competing interests.

## Funding

This work was financed by the Special Investigation on Basic Resources of Science and Technology (Grant No. 2019FY100801_03) and the Special Fund for Basic Scientific Research Business of Central Public Research Institutes of the Chinese Academy of Forestry, China (Grant No. RIF-12 and CAFYBB2017ZA004–9).

## Authors’ Contributions

T.Z., Z.Y., W.M., Q.M., and L.W. designed and conceived the study; W.M., L.L., and G.W. helped to collect the samples; T.Z., Z.Y., L.L., Q.M., and L.W. performed the experiments; T.Z., W.M., Z.Y., Q.M., X.C., and L.W. wrote and revised the manuscript. All authors read and approved the manuscript.

## Supplementary Material

giab027_GIGA-D-20-00312_Original_Submission

giab027_GIGA-D-20-00312_Revision_1

giab027_Response_to_Reviewer_Comments_Original_Submission

giab027_Reviewer_1_Report_Original_Submission Kelly Vining -- 12/9/2020 Reviewed

giab027_Reviewer_2_Report_Original_Submission Jarkko Salojarvi, DSc (tech) -- 1/27/2021 Reviewed

giab027_Supplemental_Figures_and_Tables
