# Supplementary material for: A chromosome-level reference genome of the hazelnut, Corylus heterophylla Fisch
Source: Gigascience. 2021 Apr 19;10(4):giab027. doi: 10.1093/gigascience/giab027 (PMC8054262; doi:10.1093/gigascience/giab027)
Supplement: giab027_GIGA-D-20-00312_Original_Submission [file giab027_giga-d-20-00312_original_submission.pdf]

GigaScience

A chromosome-level reference genome of the hazelnut, Corylus heterophylla Fisch.

--Manuscript Draft--

Manuscript Number:	GIGA-D-20-00312
Full Title:	A chromosome-level reference genome of the hazelnut, Corylus heterophylla Fisch.
Article Type:	Data Note
Funding Information:	Special Investigation on Basic Resources of Science and Technology (2019FY100801_03) — Not applicable<br>Basic Scientific Research Business of Central Public Research Institutes of the Chinese Academy of Forestry, China (RIF-12 and CAFYBB2017ZA004-9) — Not applicable
Abstract:	Background: Corylus heterophylla Fisch. is a species of the Betulaceae family native to China and is an economically and ecologically nut tree that can withstand cold conditions. To deepen our knowledge of Betulaceae species and facilitate the use of C. heterophylla for breeding and its genetic improvement, we have sequenced the whole-genome of C. heterophylla.<br>Findings: Based on over 64.99 Gb (~175.31 x) of nanopore long reads, we assembled a 370.75 Mb C. heterophylla genome with contig N50 and scaffold N50 sizes of 2.01 Mb and 31.33 Mb, respectively, accounting for 99.2 % of the estimated genome size. Furthermore, 361.8 Mb contigs were anchored to 11 chromosomes using Hi-C links data, representing 97.62% of the assembled genome sequences. Transcriptomes representing four different tissues were sequenced to assist protein-coding gene prediction. A total of 27,591 protein-coding genes were identified, of which 92.2% (25,389) were functionally annotated. The phylogenetic analysis showed that C. heterophylla is closed to Ostrya japonica , and diverged from their common ancestor approximately 52.79 million years ago.<br>Conclusions: We generated a high-quality chromosome-level genome of C. heterophylla. This genome resource should promote research on the molecular mechanism of hazelnut responsing to environmental stress and serve as a resource for genome-assisted improvement in cold and drought resistance of Corylus genus.
Corresponding Author:	Lujun Wang<br>Anhui Academy of Forestry<br>Hefei, CHINA
Corresponding Author Secondary Information:	
Corresponding Author's Institution:	Anhui Academy of Forestry
Corresponding Author's Secondary Institution:	
First Author:	Tiantian Zhao
First Author Secondary Information:	
Order of Authors:	Tiantian Zhao<br>Wenxu Ma<br>Zhen Yang<br>Lisong Liang<br>Guixi Wang<br>Qinghua Ma<br>Lujun Wang
Order of Authors Secondary Information:	

Powered by Editorial Manager® and ProduXion Manager® from Aries Systems Corporation

Additional Information:	
**Question**	**Response**
Are you submitting this manuscript to a special series or article collection?	No
**Experimental design and statistics**<br><br>Full details of the experimental design and statistical methods used should be given in the Methods section, as detailed in our Minimum Standards Reporting Checklist. Information essential to interpreting the data presented should be made available in the figure legends.<br><br>Have you included all the information requested in your manuscript?	Yes
**Resources**<br><br>A description of all resources used, including antibodies, cell lines, animals and software tools, with enough information to allow them to be uniquely identified, should be included in the Methods section. Authors are strongly encouraged to cite Research Resource Identifiers (RRIDs) for antibodies, model organisms and tools, where possible.<br><br>Have you included the information requested as detailed in our Minimum Standards Reporting Checklist?	Yes
**Availability of data and materials**<br><br>All datasets and code on which the conclusions of the paper rely must be either included in your submission or deposited in publicly available repositories (where available and ethically appropriate), referencing such data using a unique identifier in the references and in the "Availability of Data and Materials" section of your manuscript.	Yes

Have you have met the above requirement as detailed in our Minimum Standards Reporting Checklist?	

Powered by Editorial Manager® and ProduXion Manager® from Aries Systems Corporation

Manuscript
Click here to access/download;Manuscript;A chromosome-Giga.docx

A chromosome-level reference genome of the hazelnut, *Corylus heterophylla* Fisch.

Tiantian Zhao[1,3], Wenxu Ma[1,3], Zhen Yang[1,3], Lisong Liang[1,3], Guixi Wang[1,3], Qinghua Ma[1,3]*, Lujun Wang [2,3]*

[1]Research Institute of Forestry, Chinese Academy of Forestry/Key Laboratory of Tree Breeding and Cultivation of the State Forestry and Grassland Administration, No.1 Dongxiaofu, Xiangshan Road, Haidian District, Beijing 100091, China; [2]Anhui Academy of Forestry, No. 820 Changjiangxi Road, Shushan District, Hefei 230031, China; [3]National Hazelnut Industry Innovation Alliance/Hazelnut Engineering and Technical Research Center of the State Forestry and Grassland Administration, Xiangshan Road, Haidian District, Beijing 100091, China

*Correspondence address: Qinghua Ma, Research Institute of Forestry, Chinese Academy of Forestry, No.1 Dongxiaofu, Xiangshan Road, Haidian District, Beijing 100091, China. E-mail: mqhmary@sina.com; Lujun Wang, Anhui Academy of Forestry, No. 820 Changjiangxi Road, Shushan District, Hefei 230031, China. E-mail: wanglujun1984@163.com

Abstract

Background: *Corylus heterophylla* Fisch. is a species of the Betulaceae family native to China and is an economically and ecologically nut tree that can withstand cold conditions. To deepen our knowledge of Betulaceae species and facilitate the use of *C. heterophylla* for breeding and its genetic improvement, we have sequenced the whole-genome of *C. heterophylla*.

Findings: Based on over 64.99 Gb (~175.31 x) of nanopore long reads, we assembled a 370.75 Mb *C. heterophylla* genome with contig N50 and scaffold N50 sizes of 2.01 Mb and 31.33 Mb, respectively, accounting for 99.2 % of the estimated genome size. Furthermore, 361.8 Mb contigs were anchored to 11 chromosomes using Hi-C links data, representing 97.62% of the assembled genome sequences. Transcriptomes representing four different tissues were sequenced to assist protein-coding gene prediction. A total of 27,591 protein-coding genes were identified, of which 92.2% (25,389) were functionally annotated. The phylogenetic analysis showed that *C. heterophylla* is closed to *Ostrya japonica*, and diverged from their common ancestor approximately 52.79 million years ago.

Conclusions: We generated a high-quality chromosome-level genome of *C. heterophylla*. This genome resource should promote research on the molecular mechanism of hazelnut responsing

to environmental stress and serve as a resource for genome-assisted improvement in cold and drought resistance of *Corylus* genus.

Background

The *Corylus* genus, a member of the birch family Betulaceae and an important economically and ecologically nut tree species, is widely distributed throughout temperate regions of the Northern Hemisphere [1]. As an important nut crop, hazelnut provide the predominant flavor in a variety of cakes, candies, chocolate spreads, and butters. There are high content of unsaturated fatty acids and several essential vitamins in hazelnut oil.

The number of *Corylus* species recognized by the taxonomists ranged from 7 to 25, depending on different morphological and molecular classification [2, 3]. Among these, the European hazelnut, *Corylus avellana* L., is the species of most widely commercial cultivation with more than 400 cultivars have been described [4]. Commercial cultivation of *C. avellana* is limited to regions with climates moderated by large bodies of water that have cool summers, mild and humid winters, such as the slopes on the Black Sea of Turkey or the Willamette Valley of Oregon [5, 6]. Inadeqeate cold hardiness is a major factor limiting the expansion of commercial production into northern and inland areas. When *C. avellana* was introduced into China, it was observed that twigs withered and died almost every year in winter due to the cold, windy and dry climate in northern China. In southern China, however, the trees of European hazelnut seemed to grow well but actually bore few nuts, and abortive kernels were observed in high frequency.

Eight species and two botanical varieties of *Corylus* are reported to be native to China [5]. Among the 1.67 billion ha of wild *Corylus* in China, *Corylus heterophylla* occupies 90% of the area, which is one of the most economically wild *Corylus* species [7]. Wild *Corylus heterophylla* is mainly distributed in the mountains from northern to northeastern China. The geographical distribution range is 36.78-51.73 (°N) and 100.57-132.20 (°E), where the main climate type belongs to temperate climate. Compared with *C. avellana*, *C. heterophylla* can be adapted to regions with low temperature (-30 to -40 ℃) and drought conditions. Therefore, the characteristics of cold and drought resistance of *C. heterophylla* can be used as parent materials

for cross breeding with other hazel species.

In the present study, to better understand the molecular mechanism of hazelnut response to environmental stress, we assembled a high quality genome of *C. heterophylla* using a combination of the Oxford Nanopore high-throughput sequencing technology and the high-throughput chromosome conformation capture (Hi-C) technique. Long reads were *de novo* assembled into 1,291 polished contigs with a total size of 370.75 Mb and contig N50 and scaffold N50 values of 2.01 Mb and 31.33 Mb, respectively, which is in line with genome sizes estimated using flow cytometry and the k-mer analysis. A total of 361.8 Mb contigs were anchored into 11 chromosomes, representing 97.62% of assembled genome. Our results provide the high-quality, chromosome-level genome assembly of the *C. heterophylla*, which will support breeding programs leading to genetic improvement of hazelnuts. Furthermore, it will facilitate understanding of the special position of *Corylus* and Betulaceae in plant evolution.

Data Description

Samples collection.

Fresh and health leaves were collected from a single wild *C. heterophylla* tree in Yanqing, Beijing, China (N: 40° 32′ 27″; E: 116° 03′ 52″; Fig. 1). The fresh leaves tissue was flash frozen in liquid nitrogen for 30 min and then stored at -80 °C. DNA was extracted from leaf tissues following a previously published protocol [8]. Different tissues including root, stem, male inflorescence and leaf were sampled and flash frozen in liquid nitrogen for total RNA sequencing. Total RNA was extracted using the modified CTAB method [9].

Library preparation and whole genome sequencing.

Genomic DNA library construction was isolated from leaf tissues using DNeasy Plant Mini Kit (Qiagen) according to the manufacturer's instructions. DNA concentrations and quality were measured using NanoDrop 2000 (Thermo) and Qbit Fluorometer (Thermo Fisher), respectively. The gDNA was sheared to ~500 bp fragments using an S2 Focused-Ultrasonicator (Covaris Inc., MA, USA). Paired-end libraries (PE) were prepared using the TruSeq DNA PCR-Free Library Preparation Kit (Illumina, San Diego, CA, USA) according to the Illumina standard protocol. After quality control by Agilent 2100 Bioanalyzer and qPCR, all PCR-free libraries were

sequenced on an Illumina X-Ten platform (Illumina, San Diego, CA, USA) with 350 bp paired-end sequencing strategy according to the manufacturer's instruction. A total of 38.02 Gb (~101.76 fold coverage) clean reads were generated for genome survey and Nanopore genome polishing (Additional Table S1a).

Estimation of genome size and heterozygosity analysis.

Before genome assembly, we estimated the *C. heterophylla* genome's size using Jellyfish [10] (https://github.com/gmarcais/Jellyfish) with an optimal k-mer size. A total of 38.02 Gb short reads (~102.55 x) were processed by Jellyfish to assess their k-mer distribution (k-mer value = 19). Theoretically, the k-mer frequency follows a Poisson distribution. We selected k = 19 for the genome size estimation in this study. Genome sizes were calculated from the following equation: Genome size = 19-mer number / 19-mer depth, where 19-mer number is the total counts of each unique 19-mer and 19-mer depth is the highest frequency that occurred (Additional Fig. S1). The estimated genome size of *C. heterophylla* is 373.61 Mb.

Nanopore, RNA and Hi-C sequencing

Genomic DNA was extracted and sequenced following the instructions of the Ligation Sequencing Kit (Nanopore, Oxfordshire, UK). DNA quality was assessed by agarose gel electrophoresis and NanoDrop 2000c spectrophotometry, followed by Termo Fisher Scientifc Qubit fuorometry. After quality control, genomic DNA was size-selected using Blue Pippin BLF7510 cassette (Sage Science, Beverly, MA, USA). Libraries (fragments > 20 kb) were prepared using Oxford Nanopore Technologies' standard Ligation Sequencing kit (SQK-LSK109) protocol and sequenced on the GridION X5 platform (Oxford Nanopore, Oxford, UK) with FLOMIN106 (R9.4) flow cells. Raw ONT reads (fastq) were extracted from base-called FAST5 files using poretools [11] (https://github.com/arq5x/poretools). Then, the short reads (<5 kb) and reads having low-quality bases and adapter sequences (YSFRI, 2019c) were removed. A total of 64.99 Gb (~175.31 fold coverage) nanopore long reads with a N50 length of 27.17 kb were produced for genome assembly (Additional Fig. S2, Additional Tables S1b and S1c).

Different tissues including leaf, stem, root and male inflorescence were harvested and flash

frozen in liquid nitrogen for total RNA sequencing. The sample was subjected to poly(A) purification using oligo-dT beads (Life Technologies) followed by rRNA removal using Ribo-Zero Kit (Epicenter). The RNA quality was measured by 2100 RNA Nano 6000 Assay Kit (Agilent Technologies) and pooling together. The resulting RNA sample was used for cDNA libraries construction using the NEBNext Ultra RNA Library Prep Kit for Illumina (Neb). The quantified libraries were then prepared for sequencing on the Illumina HiSeq X-Ten system, producing 38.02 Gb paired-end reads (Additional Table S1d).

Hi-C experiments were performed essentially as described with some modifications [12, 13]. Briefly, 2g freshly harvested leaves were cut into 2- to 3-mm pieces and infiltrated in 2% formaldehyde, and crosslinking was stopped by adding glycine. The tissue was ground to powder and suspended in nuclei isolation buffer to obtain a nuclei suspension. The procedure for the Hi-C experiment, including chromatin digestion, labelling of DNA ends, DNA ligation, purification and fragmentation, was as described previously [14]. The cross-linked DNA was digested with HindIII as previously described, marked by incubating with Klenow enzyme and biotin-14-dCTP overnight at 37 ºC [14]. The 5’ overhang of the fragments was repaired and labeled using biotinylated nucleotides, followed by ligation with T4 DNA polymerase. After reversal of crosslinking, ligated DNA was purified and sheared to 300-700 bp fragments using an S2 Focused-Ultrasonicator (Covaris Inc., MA, USA). The linked DNA fragments were enriched with streptavidin beads and prepared for Illumina HiSeq X-Ten sequencing, producing 231.31 Mb (totaliing of ~69.11 Gb) Hi-C links data (Additional Table S1e).

De novo genome assembly and pseudo-chromosome construction

After the self-error correction using error correction model in Canu (version 1.5) [15], the Nanopore long reads were assembled into contigs using Wtdbg2 (version1.0) [16]. Two rounds of consensus correction were performed using Racon [17] (version 1.32) with corrected nanopore long reads, and the resulting assembly was further polished using Pilon (version 1.21) [18] with 38.02 Gb Illumina short reads (Additional Table S1a). The assembled length of 1,291 contigs of *C. heterophylla* is 370.7 Mb, accounting for 99.2 % of the estimated genome size (373.61 Mb).The contig N50 and N90 were 2.11 Mb and 138.6 kb, respectively.

The pseudo-chromosomes were constructed using Hi-C links data. The clean Hi-C reads were

mapped to the consensus contigs using the Burrows-Wheeler Aligner[19] (BWA version 0.7.17), and only uniquely mapped read pairs were considered as high quality read pairs in Hi-C analysis. The reads were removed if the mapped positions in the reference genome are out of 500 bp distance to the nearest restriction enzyme site. The quality assessment and normalization were performed using HiC-Pro[20]. There were 109,306,012 uniquely mapped PE reads, of which 58.33% (63,755,940) uniquely mapped reads were considered as valid interaction pairs for chromosome construction (Additional Table S2). The contigs were then clustered, ordered, and oriented into 11 pseudo-chromosomes using LACHESIS [20] (version 2e27abb). Finally, we obtained a high-quality chromosome-level reference genome with a total size of 370.75 Mb. The contig N50 and scaffold N50 values of were 2.01 Mb and 31.33 Mb, respectively (Table 1). A total of 361.8 Mb contigs were anchored into 11 chromosomes, representing 97.62% of assembled genome (Table 2).

Genome quality assessment

Genome completeness was assessed using the plants dataset of the Benchmarking Universal Single-Copy Orthologs (BUSCO) database (version 1.22) [21], with *e*-value < $1e^{-5}$. It detected 93.47% and 1.18%% of complete and partial gene models in *C. heterophylla* assembly results, respectively (Table 3). The core eukaryotic gene-mapping approach (CEGMA)[22] provides a method to rapidly assess genome completeness because it comprises a set of highly conserved, single-copy genes, present in all eukaryotes, containing 458 core eukaryotic genes (CEGs). We identified CEGs by CEGMA (version 2.3) pipeline [22] and found that 430 (93.89%) CEGs could be found in the assembly results (Additional Table S3a). The paired-end short libraries including 103,392,992 paired reads were remapped to the assembly genome with BWA mem[23] to assess the completeness of assembly results. More than 98.47 % of these reads could be accurately mapped into genome sequences (Additional Table S3b). Additionally, the heatmap of Hi-C interaction frequency was selected to visually assess the assembled accuracy of the *C. heterophylla* genome. The interaction heatmap was showed at 100 kb resolution. LG01-LG11 represent the eleven chromosomes of *C. heterophylla* genome, which ordered as the chromosome length. The horizontal and vertical coordinates represent the order of each ‘bin’ on the corresponding chromosome. The signal intensities clearly divided the ‘bins’ into eleven

distinct groups (LG01-LG11), demonstrating the high quality of the chromosome assignment (Fig. 2). These observations suggested the high quality and completeness of chromosome-level reference genome for *C. heterophylla*.

Repetitive elements and Protein-coding gene annotation

Repetitive elements in the *C. heterophylla* genome were identified using a combined strategy of *de novo* and homology-based approaches at the DNA and protein levels. Tandem repeats were annotated using Tandem Repeat Finder (TRF). A repeat library was constructed using MITE-Hunter [24] , LTR-FINDER (version 1.05) [25], RepeatScout (version 1.0.5) [26] and PILER [27] for *de novo* repeat content annotation. The *de novo* repeat library was classified through PASTEClassifer (version 1.0) package [28] with default parameter, and then integrated with Repbase (19.06) [29] to build a new repeat library. Finally, RepeatMasker (version 4.0.6) [30] with parameters of "-nolow -no_is -norna -engine wublast") was selected to identify and classify the genomic repetitive elements of *C. heterophylla.* In total, 210.26 Mb repetitive sequences were identified, accounting for 56.71% of *C. heterophylla* genome sequences (Table 3). The top three classed of repetitive elements were ClassI/LARD, ClassI/LTR/Gypsy and ClassI/LTR/Copia, occupying 20.51%, 11.14% and 10.44% of assembled genome sequences, respectively (Table 3).

Gene annotation was performed using a combination of ab initio prediction, homology-based gene prediction, and transcript evidence from RNA-seq data The *de novo* approach was implemented using Augustus (version 2.4) [31], Geneid [32], GlimmerHMM [33], Genscan [34] and SNAP [35]. For homology-based prediction, TBLASTN v2.2.31 [36] was used to align predicted protein sequences of *Arabidopsis thaliana*, *Betula pendula*, *Juglans regia* and *Ostrya chinensis* to the *C. heterophylla* genome with an E-value threshold of 1E-05. Then, GeMoMa (version 1.3.1) [37] was employed for homology-based gene prediction. The transcriptome data from pooled tissues of leaf, stem, root, male inflorescence of *C. heterophylla* were assembled into unigenes using Hisat (version 2.0.4) [38] and Stringtie (version 1.2.3) [39]. Then unigenes were used to predict gene structures using TransDecoder (version 2.0, http://transdecoder.github.io) [40], GeneMarkS-T (version 5.1) [41], PASA (version 2.0.2) [41]. Finally, the gene models obtained from above three approaches were integrated into a consensus

gene set using EVidenceModeler (version 1.1.0) [42] with default parameters. PASA (version 2.0.2) [43] was then used to annotate the gene structures including UTRs and alternative-splice sites (Additional Fig. S3, Additional Table S4a). A total of 27,591 non-redundant protein-coding genes were predicted for *C. heterophylla* genome (Table 1). Gene models were annotated by homologous searching against several databases using BLASTP from BLAST+ package [36] (E-value = 1e-5), including NR [44], KOG [45], TrEMBL [46] and KEGG [47] (http://www.genome.jp/kegg/) databases. InterProScan (version 4.3) [48] was used to annotated the protein motifs and domains. Blast2GO [49, 50] pipeline was used to obtain GO terms annotation from the NCBI NR database. In total, 25,389 protein coding genes (92.2%) were successfully assigned into corresponding functions (Additional Table S4b).

Whole genome-wide pseudogene identification was carried out for *C. heterophylla*. Only candidate pseudogene containing frame shifts and/or premature stop codons in its coding region were considered as a reliable pseudogene. Proteins of *C. heterophylla* were aligned to the reference genome using GenBlastA (version 1.0.4) [51] to detect the candidate homologue region. Then the candidate pseudogenes were identified using GeneWise (version 2.4.1) [52]. Finally, 2,988 pesudogenes were identified in *C. heterophylla* genome sequences (Table 1).

Different types of non-coding RNA in the *C. heterophylla* genome were identified and classified as family and subfamily. The tRNAscan-SE [53] (version 1.23) was applied to detect tRNAs. The miRNA were identified by homolog searching miRBase (Release 21) [54] against *C. heterophylla* genome with 1 mismatch. Then second structures of the putative sequences were further predicted by miRDeep2 [55]. Finally, putative miRNAs with hairpin structure were considered as reliable ones. Other types of non-coding RNA were detected using Infernal [56] (e value <= 0.01) based on Rfam databse (release 12.0) [57]. In total, 92 miRNAs: microRNAs, 617 tRNAs: transfer RNAs and 622 rRNA: ribosome RNA were annotated in *C. heterophylla* genome sequences (Additional Table S4c).

Gene family identification and phylogenetic tree construction

In the gene family and phylogenetic analysis, the protein-coding genes of *Oryza sativa*, *Arabidopsis thaliana*, *Populus trichocarpa*, *Quercus variabilis*, *Juglans regia*, *Betula pendula*, *Ostrya japonica* and *C. heterophylla* were downloaded from Genebank or Ensembl database.

The longest transcript was selected to represent the protein-coding gene. Protein sequences clustering was performed using OrthoMCL v2.0.9 [58] with default parameters to identify the gene families. The result shows that *C. heterophylla* has totaling of 16,811 gene families, including 5,150 single copy genes, 6,040 multiple copies genes and 582 specific genes. Notably, 222 species-specific families were identified for *C. heterophylla*, which may contribute to its unique features (Fig. 3A). To construct the phylogenetic analysis, 1,182 single copy orthologs were identified from one copy families of selected species. The protein sequences of single-copy orthologs were aligned by MUSCLE v3.8.31 [59], and removed low quality alignment region by Gblocks v0.91b [60] with default parameter. A phylogenetic tree was constructed with the maximum-likelihood method with the JTT amino acid substitution model implemented in the PhyML v3.3 package [61]. The divergence time was estimated using the MCMCtree program in PAML v4.7b (Phylogenetic Analysis of ML) package [62]. We used an age of (51.2 - 66.7 Mya) to calibrate the crown nodes of family Betulaceae [63]. The calibrated time (152 - 160 Mya) of *O. sativa* vs *P. trichocarpa* getting from TimeTree database was also used for divergence time estimation [64]. The result shows that *C. heterophylla* is closed to *O. japonica*, and diverged from their common ancestor at ~52.79 million years (Fig. 3B).

Conclusion

To our knowledge, this is the first report of the chromosome-level genome assembly of *C. heterophylla* using the third-generation sequencing technology of Nanopore and Hi-C. It has 210.26 Mb repetitive sequences, accounting for 56.71% of genome sequences. A total of 27,591 high-quality protein-coding genes were annotated by integrating evidences of de novo prediction, homologous protein prediction and transcriptome data. Phylogenetic analysis showed that *Corylus* is closely related to *Ostrya* and diverged from their common ancestor at approximate 52.79 Mya. This work provides valuable chromosome-level genomic data for studying loquat traits. The genomic data should promote research on the molecular mechanism of hazelnut response to environmental stress and provides valuable resource for genome-assisted improvement in *Corylus* breeding.

Additional Files

Additional Figure S1: Genome survey analysis of *C. heterophylla* based on k-mer = 19.

Additional Figure S2: Fragment size distribution of Hi-C read pairs.

Additional Figure S3: Venn plot of predicted genes generated from ab initio, RNAseq and homology methods.

Additional Table S1a. Summary of illumina data for genome survey and genome polishing.

Additional Table S1b: Statistic of Nanopore long reads.

Additional Table S1c: Distribution of length of Nanopore long reads.

Additional Table S1d: Summary of pooled transcriptome data used for gene prediction.

Additional Table S1e: Summary of Hi-C data for error correction and chromosome construction.

Additional Table S2: Valid interaction pairs of Hi-C sequencing data.

Additional Table S3a: Completeness analysis based on CEG database.

Additional Table S3b: Genome completeness assessment based on illumina sequencing reads.

Additional Table S4a: Summary of gene prediction resulted from different evidences.

Additional Table S4b: Gene function annotated by different databases.

Additional Table S4c: Non-coding RNA identification.

Abbreviations

BLAST: Basic Local Alignment Search Tool; bp: base pairs; BUSCO: Benchmarking Universal Single-Copy Orthologs; BWA: Burrows-Wheeler Aligner; CEGMA: Core Eukaryotic Genes Mapping Approach; CTAB: Hexadecyltrimethy Ammonium Bromide; Gb: gigabase pairs; GeMoMa: Gene Model Mapper; GO: Gene Ontology; Hi-C: highthroughput chromosome conformation capture; HiSeq: highthroughput sequencing; HMM: hidden Markov model; kb: kilobase pairs; KEGG: Kyoto Encyclopedia of Genes and Genomes; KOG: EuKaryotic Orthologous Groups; LG: linkage group; LTR: long terminal repeat; Mb: megabase pairs; miRNA: microRNA; MITE: miniature inverted-repeat transposable element; MUSCLE: MUltiple Sequence Comparison by Log-Expectation; Mya: million years ago; NCBI: National Center for Biotechnology Information; NR: non-redundant; PAML: Phylogenetic Analysis of Maximum-Likelihood; PASA: Program to Assemble Spliced Alignments; PCR: polymerase chain reaction; PE: paired-end; PhyML: Phylogeny Maximum Likelihood; RNA-seq: RNA

sequencing; rRNA: ribosomal RNA; SAAS: Shanghai Academy of Agricultural Sciences; SNAP: Semi-HMM-based Nucleic Acid Parser; TIR: terminal inverted repeat; TrEMBL: a database of translated proteins from European Bioinformatics Institute; TRF: Tandem Repeat Finder; tRNA: transfer RNA.

Competing Interests

The authors declare that they have no competing interests.

Funding

This work was financed by the Special Investigation on Basic Resources of Science and Technology (Grant No. 2019FY100801_03) and the Special Fund for Basic Scientific Research Business of Central Public Research Institutes of the Chinese Academy of Forestry, China (Grant No. RIF-12 and CAFYBB2017ZA004-9).

Authors' Contributions

T.Z., Z.Y., W.M., Q.M., and L.W. designed and conceived the study; W.M., L.L., and G.X. helped to collect the samples; T.Z., Z.Y., L.L., Q.M., and L.W. performed the experiments; T.Z., W.M., Z.Y., Q.M., and L.W. wrote and revised the manuscript. All authors read and approved the manuscript.

Availability of supporting data

The genome sequence data has been deposited in NCBI under the accession xx. The version described in this paper is version xx. Raw reads of Nanopore, WGS, Hi-C and RNAseq, and genome assembly sequences of the *C. heterophylla* genome have been deposited at the Genome Sequence Archive in NCBI under BioProject PRJNA655406 and BioSample Accessions of SAMN15734705 and SAMN15734794. All supplementary figures and tables are provided in Additional Files. Supporting data including annotations and RNA-seq data and phylogenetic trees are available in the GigaDB database (ref).

Acknowledgements

We thank Changmian Ji from Chinese Academy of Tropical Agricultural Sciences for assistant of bioinformatic analysis and discussion.

References

1. Zong JW, Zhao TT, Ma QH, Liang LS and Wang GX. Assessment of Genetic Diversity and Population Genetic Structure of Corylus mandshurica in China Using SSR Markers. PLoS One. 2015;10 9:e0137528. doi:10.1371/journal.pone.0137528.
2. Mehlenbacher SA. Hazelnuts. A guide to nut tree culture in north america. Northern Nut Growers Association, Inc; 2003.
3. Boccacci P, Beltramo C, Sandoval Prando MA, Lembo A, Sartor C, Mehlenbacher SA, et al. In silico mining, characterization and cross-species transferability of EST-SSR markers for European hazelnut (Corylus avellana L.). Molecular Breeding. 2015;35 1:21. doi:10.1007/s11032-015-0195-7.
4. Gürcan K, Mehlenbacher S, Botta R and Boccacci P. Development, characterization, segregation, and mapping of microsatellite markers for European hazelnut (Corylus avellana L.) from enriched genomic libraries and usefulness in genetic diversity studies. Tree Genetics & Genomes. 2010;6 4:513-31.
5. ZHANG Yuhe LL, LIANG Weijian,ZHANG Yuming. China fruit's monograph-chestnut and hazelnut. Beijing:China Forestry Publishing House; 2005.
6. Molnar TJ. Corylus. Wild Crop Relatives: Genomic and Breeding Resources. 1 ed. Forest Trees: Springer-Verlag Berlin Heidelberg; 2011.
7. Wang GX, Ma QH, Zhao TT and Liang LS. Resources and production of hazelnut in China. Acta horticulturae. 2018; 1226:59-64.
8. Mayjonade B, Gouzy J, Donnadieu C, Pouilly N, Marande W, Callot C, et al. Extraction of high-molecular-weight genomic DNA for long-read sequencing of single molecules. Biotechniques. 2016;61 4:203-5. doi:10.2144/000114460.
9. Doyle J. A rapid DNA isolation procedure for small quantities of fresh leaf tissue. Phytochem Bull. 1987;19.
10. Marçais G and Kingsford C. A fast, lock-free approach for efficient parallel counting of occurrences of k-mers. Bioinformatics. 2011;27 6:764-70. doi:10.1093/bioinformatics/btr011.
11. Loman NJ and Quinlan AR. Poretools: a toolkit for analyzing nanopore sequence data. Bioinformatics. 2014;30 23:3399-401. doi:10.1093/bioinformatics/btu555.
12. Belton JM, McCord RP, Gibcus JH, Naumova N, Zhan Y and Dekker J. Hi-C: a comprehensive technique to capture the conformation of genomes. Methods. 2012;58 3:268-76. doi:10.1016/j.ymeth.2012.05.001.
13. Grob S, Schmid MW and Grossniklaus U. Hi-C analysis in Arabidopsis identifies the KNOT, a structure with similarities to the flamenco locus of Drosophila. Mol Cell. 2014;55 5:678-93. doi:10.1016/j.molcel.2014.07.009.
14. Xie T, Zheng JF, Liu S, Peng C, Zhou YM, Yang QY, et al. De novo plant genome assembly based on chromatin interactions: a case study of Arabidopsis thaliana. Mol Plant. 2015;8 3:489-92. doi:10.1016/j.molp.2014.12.015.
15. Koren S, Walenz BP, Berlin K, Miller JR, Bergman NH and Phillippy AM. Canu: scalable and accurate long-read assembly via adaptive k-mer weighting and repeat separation. Genome Res. 2017;27 5:722-36. doi:10.1101/gr.215087.116.

16. Ruan J and Li H. Fast and accurate long-read assembly with wtdbg2. Nat Methods. 2020;17 2:155-8. doi:10.1038/s41592-019-0669-3.
17. Vaser R, Sović I, Nagarajan N and Šikić M. Fast and accurate de novo genome assembly from long uncorrected reads. Genome Research. 2017;27 5:737-46. doi:10.1101/gr.214270.116.
18. Walker BJ, Abeel T, Shea T, Priest M, Abouelliel A, Sakthikumar S, et al. Pilon: an integrated tool for comprehensive microbial variant detection and genome assembly improvement. PLoS One. 2014;9 11:e112963. doi:10.1371/journal.pone.0112963.
19. Li H and Durbin R. Fast and accurate short read alignment with Burrows-Wheeler transform. Bioinformatics. 2009;25 14:1754-60. doi:10.1093/bioinformatics/btp324.
20. Burton JN, Adey A, Patwardhan RP, Qiu R, Kitzman JO and Shendure J. Chromosome-scale scaffolding of de novo genome assemblies based on chromatin interactions. Nat Biotechnol. 2013;31 12:1119-25. doi:10.1038/nbt.2727.
21. Seppey M, Manni M and Zdobnov EM. BUSCO: Assessing Genome Assembly and Annotation Completeness. Methods Mol Biol. 2019;1962:227-45. doi:10.1007/978-1-4939-9173-0_14.
22. Parra G, Bradnam K and Korf I. CEGMA: a pipeline to accurately annotate core genes in eukaryotic genomes. Bioinformatics. 2007;23 9:1061-7. doi:10.1093/bioinformatics/btm071.
23. Li H and Durbin R. Fast and accurate long-read alignment with Burrows-Wheeler transform. Bioinformatics. 2010;26 5:589-95. doi:10.1093/bioinformatics/btp698.
24. Han Y and Wessler SR. MITE-Hunter: a program for discovering miniature inverted-repeat transposable elements from genomic sequences. Nucleic Acids Res. 2010;38 22:e199. doi:10.1093/nar/gkq862.
25. Xu Z and Wang H. LTR_FINDER: an efficient tool for the prediction of full-length LTR retrotransposons. Nucleic Acids Res. 2007;35 Web Server issue:W265-8. doi:10.1093/nar/gkm286.
26. Price AL, Jones NC and Pevzner PA. De novo identification of repeat families in large genomes. Bioinformatics. 2005;21 Suppl 1:i351-8. doi:10.1093/bioinformatics/bti1018.
27. Edgar RC and Myers EW. PILER: identification and classification of genomic repeats. Bioinformatics. 2005;21 Suppl 1:i152-8. doi:10.1093/bioinformatics/bti1003.
28. Hoede C, Arnoux S, Moisset M, Chaumier T, Inizan O, Jamilloux V, et al. PASTEC: an automatic transposable element classification tool. PLoS One. 2014;9 5:e91929. doi:10.1371/journal.pone.0091929.
29. Bao W, Kojima KK and Kohany O. Repbase Update, a database of repetitive elements in eukaryotic genomes. Mob DNA. 2015;6:11. doi:10.1186/s13100-015-0041-9.
30. Tarailo-Graovac M and Chen N. Using RepeatMasker to identify repetitive elements in genomic sequences. Curr Protoc Bioinformatics. 2009;Chapter 4:Unit 4.10. doi:10.1002/0471250953.bi0410s25.
31. Stanke M and Morgenstern B. AUGUSTUS: a web server for gene prediction in eukaryotes that allows user-defined constraints. Nucleic Acids Res. 2005;33 Web Server issue:W465-7. doi:10.1093/nar/gki458.
32. Alioto T, Blanco E, Parra G and Guigó R. Using geneid to Identify Genes. Curr Protoc Bioinformatics. 2018;64 1:e56. doi:10.1002/cpbi.56.
33. Majoros WH, Pertea M and Salzberg SL. TigrScan and GlimmerHMM: two open source ab initio eukaryotic gene-finders. Bioinformatics. 2004;20 16:2878-9. doi:10.1093/bioinformatics/bth315.
34. Burge C and Karlin S. Prediction of complete gene structures in human genomic DNA. J Mol Biol. 1997;268 1:78-94. doi:10.1006/jmbi.1997.0951.
35. Korf I. Gene finding in novel genomes. BMC Bioinformatics. 2004;5:59. doi:10.1186/1471-2105-5-59.
36. Camacho C, Coulouris G, Avagyan V, Ma N, Papadopoulos J, Bealer K, et al. BLAST+: architecture and applications. BMC Bioinformatics. 2009;10:421. doi:10.1186/1471-2105-10-421.

37. Keilwagen J, Wenk M, Erickson JL, Schattat MH, Grau J and Hartung F. Using intron position conservation for homology-based gene prediction. Nucleic Acids Res. 2016;44 9:e89. doi:10.1093/nar/gkw092.
38. Kim D, Langmead B and Salzberg SL. HISAT: a fast spliced aligner with low memory requirements. Nat Methods. 2015;12 4:357-60. doi:10.1038/nmeth.3317.
39. Pertea M, Pertea GM, Antonescu CM, Chang TC, Mendell JT and Salzberg SL. StringTie enables improved reconstruction of a transcriptome from RNA-seq reads. Nat Biotechnol. 2015;33 3:290-5. doi:10.1038/nbt.3122.
40. TransDecoder. https://github.com/TransDecoder/TransDecoder.
41. Tang S, Lomsadze A and Borodovsky M. Identification of protein coding regions in RNA transcripts. Nucleic Acids Res. 2015;43 12:e78. doi:10.1093/nar/gkv227.
42. Haas BJ, Salzberg SL, Zhu W, Pertea M, Allen JE, Orvis J, et al. Automated eukaryotic gene structure annotation using EVidenceModeler and the Program to Assemble Spliced Alignments. Genome Biol. 2008;9 1:R7. doi:10.1186/gb-2008-9-1-r7.
43. Campbell MA, Haas BJ, Hamilton JP, Mount SM and Buell CR. Comprehensive analysis of alternative splicing in rice and comparative analyses with Arabidopsis. BMC Genomics. 2006;7:327. doi:10.1186/1471-2164-7-327.
44. Deng YY, Li JQ, Wu SF, Zhu YP and He FC. Integrated nr Database in Protein Annotation System and Its Localization. Computer Engineering. 2006;32 5:71-2.
45. Koonin EV, Fedorova ND, Jackson JD, Jacobs AR, Krylov DM, Makarova KS, et al. A comprehensive evolutionary classification of proteins encoded in complete eukaryotic genomes. Genome Biol. 2004;5 2:R7. doi:10.1186/gb-2004-5-2-r7.
46. Boeckmann B, Bairoch A, Apweiler R, Blatter MC, Estreicher A, Gasteiger E, et al. The SWISS-PROT protein knowledgebase and its supplement TrEMBL in 2003. Nucleic Acids Res. 2003;31 1:365-70. doi:10.1093/nar/gkg095.
47. Kanehisa M, Furumichi M, Tanabe M, Sato Y and Morishima K. KEGG: new perspectives on genomes, pathways, diseases and drugs. Nucleic Acids Res. 2017;45 D1:D353-d61. doi:10.1093/nar/gkw1092.
48. Zdobnov EM and Apweiler R. InterProScan--an integration platform for the signature-recognition methods in InterPro. Bioinformatics. 2001;17 9:847-8. doi:10.1093/bioinformatics/17.9.847.
49. Conesa A and Götz S. Blast2GO: A comprehensive suite for functional analysis in plant genomics. Int J Plant Genomics. 2008;2008:619832. doi:10.1155/2008/619832.
50. Götz S, García-Gómez JM, Terol J, Williams TD, Nagaraj SH, Nueda MJ, et al. High-throughput functional annotation and data mining with the Blast2GO suite. Nucleic Acids Res. 2008;36 10:3420-35. doi:10.1093/nar/gkn176.
51. She R, Chu JS, Wang K, Pei J and Chen N. GenBlastA: enabling BLAST to identify homologous gene sequences. Genome Res. 2009;19 1:143-9. doi:10.1101/gr.082081.108.
52. Birney E and Durbin R. Using GeneWise in the Drosophila annotation experiment. Genome Res. 2000;10 4:547-8. doi:10.1101/gr.10.4.547.
53. Lowe TM and Eddy SR. tRNAscan-SE: a program for improved detection of transfer RNA genes in genomic sequence. Nucleic Acids Res. 1997;25 5:955-64. doi:10.1093/nar/25.5.955.
54. Kozomara A, Birgaoanu M and Griffiths-Jones S. miRBase: from microRNA sequences to function. Nucleic Acids Res. 2019;47 D1:D155-d62. doi:10.1093/nar/gky1141.
55. Friedländer MR, Mackowiak SD, Li N, Chen W and Rajewsky N. miRDeep2 accurately identifies known and hundreds of novel microRNA genes in seven animal clades. Nucleic Acids Res. 2012;40 1:37-52. doi:10.1093/nar/gkr688.

56. Nawrocki EP and Eddy SR. Infernal 1.1: 100-fold faster RNA homology searches. Bioinformatics. 2013;29 22:2933-5. doi:10.1093/bioinformatics/btt509.
57. Nawrocki EP, Burge SW, Bateman A, Daub J, Eberhardt RY, Eddy SR, et al. Rfam 12.0: updates to the RNA families database. Nucleic Acids Res. 2015;43 Database issue:D130-7. doi:10.1093/nar/gku1063.
58. Li L, Stoeckert CJ, Jr. and Roos DS. OrthoMCL: identification of ortholog groups for eukaryotic genomes. Genome Res. 2003;13 9:2178-89. doi:10.1101/gr.1224503.
59. Edgar RC. MUSCLE: multiple sequence alignment with high accuracy and high throughput. Nucleic Acids Res. 2004;32 5:1792-7. doi:10.1093/nar/gkh340.
60. Talavera G and Castresana J. Improvement of phylogenies after removing divergent and ambiguously aligned blocks from protein sequence alignments. Syst Biol. 2007;56 4:564-77. doi:10.1080/10635150701472164.
61. Guindon S, Dufayard JF, Lefort V, Anisimova M, Hordijk W and Gascuel O. New algorithms and methods to estimate maximum-likelihood phylogenies: assessing the performance of PhyML 3.0. Syst Biol. 2010;59 3:307-21. doi:10.1093/sysbio/syq010.
62. Yang Z. PAML 4: phylogenetic analysis by maximum likelihood. Mol Biol Evol. 2007;24 8:1586-91. doi:10.1093/molbev/msm088.
63. Takhtajan AL. Outline of the classification of flowering plants (magnoliophyta). Botanical Review. 1980;46 3:225-359.
64. TimeTree database. http://www.timetree.org/.

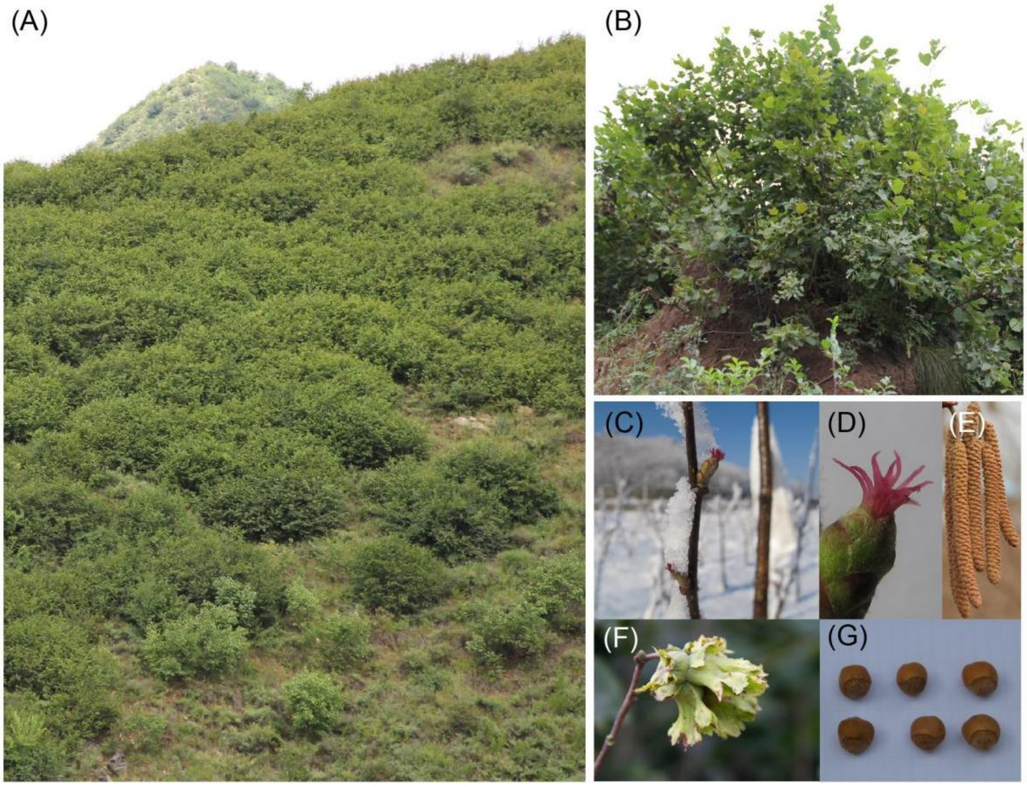

Figure1: Morphological characters of the hazelnut variety, *C. heterophylla*. Mature plants in panel (A) and (B), female inflorescence of (C) and (D), male inflorescence (E), fruit with husk (F), and nuts (C) are shown.

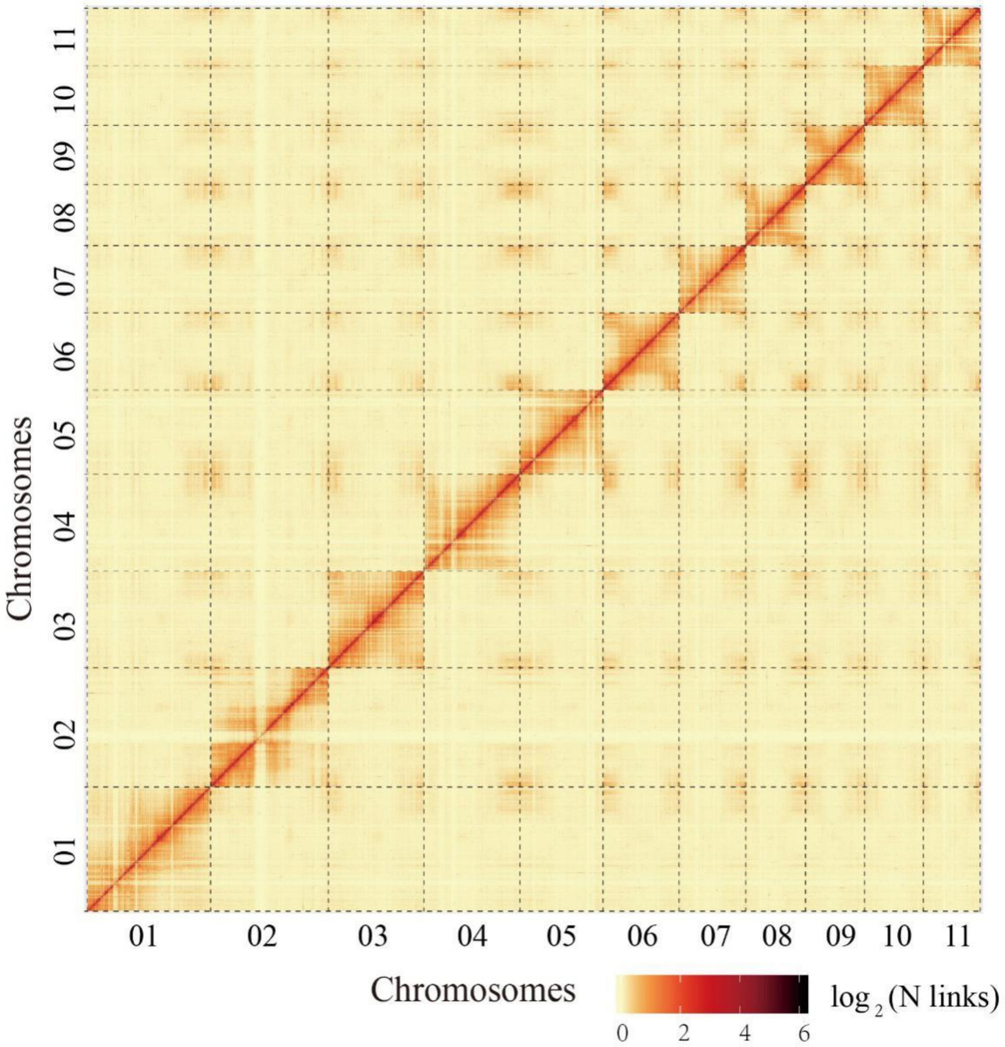

Figure2: Interaction frequency distribution of Hi-C links among eleven chromosomes. Genome-wide Hi-C map of *C. heterophylla*. We scanned the genome by 500-kb nonoverlapping window as a bin and calculated valid interaction links of Hi-C data between any pair of bins. The log2 of link number was transformed. The color key of heatmap ranging from light yellow to dark red represented the frequency of Hi-C interaction links from low to high (0~6).

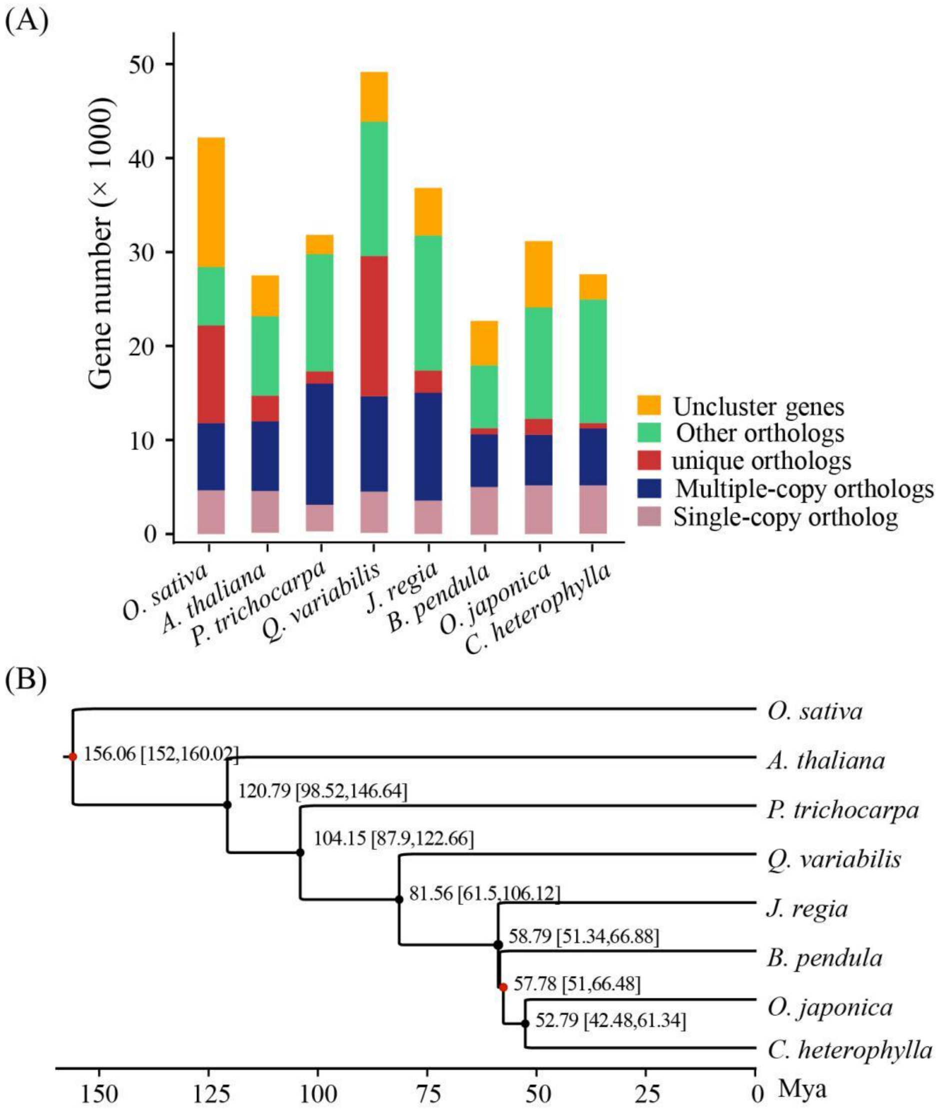

Figure3: Genome evolution analysis of *C. heterophylla*. (A) Summary of gene family clustering of *C. heterophylla* and 7 related species. Single-copy ortholog, one copy genes in ortholog group. Multiple-copy orthologs, multiple genes in ortholog group. Unique orthologs, species-specific genes. Other orthologs, the rest of the clustered genes. Uncluster genes, number of genes out of cluster. (B) Phylogenetic relationship and divergence time estimation (MYA, millions of years ago). The *O. sativa* was considered as outgroup in phylogenetic tree construction. The red dots indicate the fossil correction time of *O. sativa* vs *P. trichocarpa* (152 - 160 Mya) and crown nodes of family Betulaceae (51.2 - 66.7 Mya), respectively.

Table 1. Statistics of assembly results of *C. heterophylla* genome.

Feature	*C. heterophylla*
Genome size (bp)	370,750,808
Contig number	1,328
Maximum contig length (bp)	9,680,353
Contig N50 (bp)	2,068,510
Contig L50	48
Contig N90 (bp)	125,301
Scaffold number	951
Maximum scaffold length (bp)	46,514,939
Scaffold N50 (bp)	31,328,411
Scaffold L50	5
Scaffold N90 (bp)	21,561,575
GC content (%)	35.84
Gene number	27,591
Gene length (bp)	123,431,253
Average gene length (bp)	4,473.61
Exon number	138,886
Exon length (bp)	33,679,425
Intron number	138,885
Intron length (bp)	89,751,828
Pseudogenes	2,988
Pseudogene length (bp)	7,166,319

Note: only sequences whose length is more than 1 kb are considered.

Table 2. Summary of eleven pseudo-chromosomes for *C. heterophylla*.

Chr	No. of clustered sequences	Length of clustered sequences (bp)	No. of ordered sequences	Length of ordered sequences (bp)
LG01	114	49,577,893	56	46,509,439
LG02	113	48,019,691	49	44,425,769
LG03	67	37,395,073	33	36,016,943
LG04	95	38,562,170	53	36,392,613
LG05	85	34,656,877	37	31,324,811
LG06	76	31,263,564	31	28,814,739
LG07	103	29,494,057	36	25,003,895
LG08	45	23,716,498	23	22,749,571
LG09	41	23,427,462	17	22,292,654
LG10	41	23,093,417	25	22,249,747
LG11	53	22,694,573	28	21,558,875
Total (%)	833 (62.73)	361,901,275 (97.62)	388 (46.58)	337,339,056 (93.21)

Table 3. Genome completeness assessment by BUSCO.

Categories	Number	Percent (%)
Complete BUSCOs	1,346	93.47
Complete and single-copy BUSCOs	1,296	90.00
Complete and duplicated BUSCOs	50	3.47
Fragmented BUSCOs	17	1.18
Missing BUSCOs	77	5.35
Total BUSCO groups searched	1,440	100.00

Table 4. Repetitive elements in the *C. heterophylla* genome.

Classes	Number	Length (bp)	Percent (%)
ClassI	584,311	169,738,018	45.78
ClassI/DIRS	18,638	7,059,337	1.9
ClassI/LARD	303,288	76,033,830	20.51
ClassI/LINE	60,182	18,890,786	5.1
ClassI/LTR/Copia	101,158	38,719,023	10.44
ClassI/LTR/Gypsy	83,300	41,302,761	11.14
ClassI/LTR/Unknown	1,953	1,080,718	0.29
ClassI/PLE	5,600	4,125,513	1.11
ClassI/SINE	5,344	1,058,985	0.29
ClassI/TRIM	3,828	1,023,113	0.28
ClassI/Unknown	1,020	244,561	0.07
ClassII	77,407	24,382,510	6.58
ClassII/Crypton	455	109,226	0.03
ClassII/Helitron	27,254	8,348,317	2.25
ClassII/MITE	1,112	194,088	0.05
ClassII/Maverick	754	165,986	0.04
ClassII/TIR	44,403	15,342,483	4.14
ClassII/Unknown	3,429	459,116	0.12
PotentialHostGene	46,369	9,994,181	2.7
SSR	1,135	265,113	0.07
Unknown	116,728	26,584,597	7.17
Total	825,950	210,255,221	56.71

DIRS: dictyostelium intermediate repeat sequence; LARD: large retrotransposon derivative; LINE: long interspersed nuclear element; LTR: long terminal repeat; MITE: miniature inverted-repeat transposable element; PLE: Penelope-like element; SINE: short interspersed nuclear element; SSR: simple sequence repeat; TIR: terminal inverted repeat; TRIM: terminal-repeat retrotransposons in miniature.

Supplementary Tables

Click here to access/download
Supplementary Material
Supplementary tables.xls

Supplementary Figures

Click here to access/download

Supplementary Material

Supplementary figures.docx
