# Supplementary material for: A chromosome-level reference genome of the hazelnut, Corylus heterophylla Fisch
Source: Gigascience. 2021 Apr 19;10(4):giab027. doi: 10.1093/gigascience/giab027 (PMC8054262; doi:10.1093/gigascience/giab027)
Supplement: giab027_GIGA-D-20-00312_Revision_1 [file giab027_giga-d-20-00312_revision_1.pdf]

GigaScience

A chromosome-level reference genome of the hazelnut, Corylus heterophylla Fisch.

--Manuscript Draft--

**Manuscript Number:**	GIGA-D-20-00312R1
**Full Title:**	A chromosome-level reference genome of the hazelnut, Corylus heterophylla Fisch.
**Article Type:**	Data Note
**Funding Information:**	Special Investigation on Basic Resources of Science and Technology (2019FY100801_03) — Not applicable<br>Basic Scientific Research Business of Central Public Research Institutes of the Chinese Academy of Forestry, China (RIF-12 and CAFYBB2017ZA004-9) — Not applicable
**Abstract:**	Background: Corylus heterophylla Fisch. is a species of the Betulaceae family native to China. As an economically and ecologically important nut tree, C. heterophylla can survive in extremely low temperatures (–30 to –40 °C). To deepen our knowledge of the Betulaceae species and facilitate the use of C. heterophylla for breeding and its genetic improvement, we have sequenced the whole genome of C. heterophylla .<br>Findings: Based on over 64.99 Gb (~175.31 x) of Nanopore long reads, we assembled a 370.75 Mb C. heterophylla genome with contig N50 and scaffold N50 sizes of 2.01 Mb and 31.33 Mb, respectively, accounting for 99.2% of the estimated genome size. Furthermore, 361.8 Mb contigs were anchored to 11 chromosomes using Hi-C links data, representing 97.62% of the assembled genome sequences. Transcriptomes representing four different tissues were sequenced to assist protein-coding gene prediction. A total of 27,591 protein-coding genes were identified, of which 92.2% (25,389) were functionally annotated. The phylogenetic analysis showed that C. heterophylla is close to Ostrya japonica , and they diverged from their common ancestor approximately 52.79 million years ago.<br>Conclusions: We generated a high-quality chromosome-level genome of C. heterophylla . This genome resource will promote research on the molecular mechanisms of how the hazelnut responds to environmental stresses and serves as an important resource for genome-assisted improvement in cold and drought resistance of the Corylus genus .
**Corresponding Author:**	Lujun Wang<br>Anhui Academy of Forestry<br>Hefei, CHINA
**Corresponding Author Secondary Information:**	
**Corresponding Author's Institution:**	Anhui Academy of Forestry
**Corresponding Author's Secondary Institution:**	
**First Author:**	Tiantian Zhao
**First Author Secondary Information:**	
**Order of Authors:**	Tiantian Zhao
	Wenxu Ma
	Zhen Yang
	Lisong Liang
	Xin Chen
	Guixi Wang
	Qinghua Ma
	Lujun Wang

Powered by Editorial Manager® and ProduXion Manager® from Aries Systems Corporation

**Order of Authors Secondary Information:**	
**Response to Reviewers:**	19 February, 2021<br>Editor : Dr. Hongling Zhou<br>GigaScience<br>Ms. No. GIGA-D-20-00312<br><br>Dear Dr. Hongling Zhou,<br>We appreciate the time and effort that you and reviewers dedicated to provide the helpful feedback on our manuscript “A chromosome-level reference genome of the hazelnut, Corylus heterophylla Fisch”(GIGA-D-20-00312). We have carefully revised our manuscript in light of your extensive and helpful comments and those of the reviewers. We have added the RRID of the biological tools obtained from SciCrunch.org database into the revised manuscript. In order to make the figure more neat and appropriate, we adjusted Figure 1 (C, D) and replaced them with new photos. We also revised the corresponding figure legends of Figure 1. Here we resubmit our revised manuscript to Journal of the GigaScience. Below please find a detailed response to the questions raised by the reviewers. During the proof reading and revision of this paper, Dr. Xin Chen has given us great help, so with the consent of all the authors, we hope to add him as one of the co-authors of this paper. Finally, the revised manuscript has obtained a language editing help from Charlesworth Author Services Team. We hope the revised manuscript would satisfy you and reviewers.<br><br>Thank you again for your time and effort.<br><br>Sincerely,<br><br>Lujun Wang<br><br>Response to Reviewer #1:<br>1 Reviewer’s comment: Lines 46-50: A reference for these statements is needed.<br>Author’s response: Thanks for the reviewer helpful comments for our work. As suggested, we added reference in line 52.<br><br>2 Reviewer’s comment: Line 54: What is meant by "most economically wild"?<br>Author’s response: Thanks to reviewer’s attention. To avoid confusion, we rewrote this sentence as “Corylus heterophylla is one of the most important economic Corylus species. Among the 1.67 million ha of wild Corylus in China, C. heterophylla occupies 90% of the area.” in the revised manuscript.<br><br>3 Reviewer’s comment: Line-s 242-243: It appears that you are confusing ortholog groups with gene families. OrthoMCL is used to detect ortholog groups, not gene families.<br>Author’s response: We agree with reviewer’s opinion. We replaced the “gene families” to “ortholog groups” in the revised manuscript.<br><br>4 Reviewer’s comment: Line 290: Hexadecyltrimethyl is missing the "l" at the end.<br>Author’s response: Sorry for this spelling mistake. We have corrected this mistake in the revised manuscript.<br><br>Response to Reviewer #2:<br>1 Reviewer’s comment: The accession IDs for NCBI and SRA were still missing from this version, I would like to request that the data be deposited to the repository upon publication of the assembly.<br>Author’s response: Many thanks for reviewer’s helpful suggestion. As suggested, we have add the NCBI accession IDs for genome (JADOBO000000000) and SRA (SRR12458330, SRR12458329, SRR12458328, SRR12458327) at Availability of supporting data section (lines 346-350)<br><br>2 Reviewer’s comment: The paper needs proof reading and help from a native English speaking person. Author’s response: As reviewer’s suggestion, the paper has been send to Charlesworth Author Services Team for English language revision.<br><br>3 Reviewer’s comment: Line 16: economically and ecologically important nut tree<br>Author’s response: We have corrected this sentence in the revised manuscript.

	4 Reviewer's comment: Line 19: Nanopore (capital letter)<br>Author's response: We have corrected the "nanopore" as "Nanopore" in the revised manuscript.<br><br>5 Reviewer's comment: Line 29: bad english: molecular mechanism of hazelnut responsing to environmental stress and serve as a resource ->molecular mechanisms of how hazel nut responds to environmental stresses and serves as a resource (or similar)<br>Author's response: As reviewer's suggestion, we have corrected this sentence as "This genome resource will promote research on the molecular mechanisms of how the hazelnut responds to environmental stresses and serves as an important resource for genome-assisted improvement in cold and drought resistance of the Corylus genus" in the revised manuscript (lines 31-34).<br><br>6 Reviewer's comment: 35: provides<br>Author's response: As suggested, we have corrected the word "provide" as "provides" in the revised manuscript.<br><br>7 Reviewer's comment: 36: There is a high content<br>Author's response: We have corrected "are" as "is" in the revised manuscript.<br><br>8 Reviewer's comment: 39 ranges (or varies between)<br>Author's response: We revised this spelling mistake as "ranges" in the revised manuscript.<br><br>9 Reviewer's comment: 45: Inadequate<br>Author's response: We have revised the spelling mistake as "Inadequate" in the revise manuscript.<br><br>10 Reviewer's comment: 54: "one of the most economically wild Corylus species" - what does this mean?<br>Author's response: To avoid confusion, we have revised this sentence as "Corylus heterophylla is one of the most important economic Corylus species. Among the 1.67 million ha of wild Corylus in China, C. heterophylla occupies 90% of the area." in the revised manuscript (lines 54-55).<br><br>11 Reviewer's comment: 85 Qbit -> Qubit<br>Author's response: We revised this spelling mistake as "Qubit" in the revised manuscript.<br><br>12 Reviewer's comment: 109: fuorometry -> fluorometry<br>Author's response: We revised the spelling mistake as "fluorometry" in the revised manuscript.<br><br>13 Reviewer's comment: 253: this is the monocot - dicot split time<br>Author's response: Thanks for reviewer's comment. To avoid confusion, we revised this sentence as "The monocot-dicot split time (152 - 160 Mya) getting from TimeTree database was also used to calibrate the time estimation".
**Additional Information:**	
**Question**	**Response**
Are you submitting this manuscript to a special series or article collection?	No
**Experimental design and statistics**<br><br>Full details of the experimental design and statistical methods used should be given in the Methods section, as detailed in our Minimum Standards Reporting Checklist.	Yes

Powered by Editorial Manager® and ProduXion Manager® from Aries Systems Corporation

Information essential to interpreting the data presented should be made available in the figure legends.<br><br>Have you included all the information requested in your manuscript?	
**Resources**<br><br>A description of all resources used, including antibodies, cell lines, animals and software tools, with enough information to allow them to be uniquely identified, should be included in the Methods section. Authors are strongly encouraged to cite Research Resource Identifiers (RRIDs) for antibodies, model organisms and tools, where possible.<br><br>Have you included the information requested as detailed in our Minimum Standards Reporting Checklist?	Yes
**Availability of data and materials**<br><br>All datasets and code on which the conclusions of the paper rely must be either included in your submission or deposited in publicly available repositories (where available and ethically appropriate), referencing such data using a unique identifier in the references and in the “Availability of Data and Materials” section of your manuscript.<br><br>Have you have met the above requirement as detailed in our Minimum Standards Reporting Checklist?	Yes

Powered by Editorial Manager® and ProduXion Manager® from Aries Systems Corporation

Revised manuscript Click here to access/download;Manuscript;A chromosome-Giga-2021.2.19-v2.docx

A chromosome-level reference genome of the hazelnut, *Corylus heterophylla* Fisch.

Tiantian Zhao[1,3], Wenxu Ma[1,3], Zhen Yang[1,3], Lisong Liang[1,3], Xin Chen[3,4], Guixi Wang[1,3], Qinghua Ma[1,3]*, Lujun Wang [2,3]*

[1]Research Institute of Forestry, Chinese Academy of Forestry/Key Laboratory of Tree Breeding and Cultivation of the State Forestry and Grassland Administration, No.1 Dongxiaofu, Xiangshan Road, Haidian District, Beijing 100091, China; [2]Anhui Academy of Forestry, No. 820 Changjiangxi Road, Shushan District, Hefei 230031, China; [3]National Hazelnut Industry Innovation Alliance/Hazelnut Engineering and Technical Research Center of the State Forestry and Grassland Administration, Xiangshan Road, Haidian District, Beijing 100091, China; [4]Shandong Institute of Pomology, Shandong Academy of Agricultural Sciences, No.66 Longtan Road, Taishan District, Taian 271000, China

*Correspondence address: Qinghua Ma, Research Institute of Forestry, Chinese Academy of Forestry, No.1 Dongxiaofu, Xiangshan Road, Haidian District, Beijing 100091, China. E-mail: mqhmary@sina.com; Lujun Wang, Anhui Academy of Forestry, No. 820 Changjiangxi Road, Shushan District, Hefei 230031, China. E-mail: wanglujun1984@163.com

ORCIDs:

Tiantian Zhao: https://orcid.org/0000-0003-2700-5314

Wenxu Ma: https://orcid.org/0000-0002-0348-1167

Zhen Yang: https://orcid.org/0000-0002-6807-962X

Lisong Liang: https://orcid.org/0000-0002-0046-0168

Xin Chen: https://orcid.org/0000-0003-4538-7010

Guixi Wang: https://orcid.org/0000-0002-9949-6004

Qinghua Ma: https://orcid.org/0000-0002-6065-0852

Lujun Wang: https://orcid.org/0000-0002-8046-6067

Abstract

Background: *Corylus heterophylla* Fisch. is a species of the Betulaceae family native to China. As an economically and ecologically important nut tree, *C. heterophylla* can survive in extremely low temperatures (–30 to –40 °C). To deepen our knowledge of the Betulaceae species and facilitate the use of *C. heterophylla* for breeding and its genetic improvement, we have sequenced the whole genome of *C. heterophylla*.

Findings: Based on over 64.99 Gb (~175.30 x) of Nanopore long reads, we assembled a 370.75 Mb *C. heterophylla* genome with contig N50 and scaffold N50 sizes of 2.07 Mb and 31.33 Mb, respectively, accounting for 99.23% of the estimated genome size (373.61 Mb). Furthermore, 361.90 Mb contigs were anchored to 11 chromosomes using Hi-C links data, representing 97.61% of the assembled genome sequences. Transcriptomes representing four different tissues were sequenced to assist protein-coding gene prediction. A total of 27,591 protein-coding genes were identified, of which 92.02% (25,389) were functionally annotated. The phylogenetic analysis showed that *C. heterophylla* is close to *Ostrya japonica*, and they diverged from their common ancestor approximately 52.79 million years ago.

Conclusions: We generated a high-quality chromosome-level genome of *C. heterophylla*. This genome resource will promote research on the molecular mechanisms of how the hazelnut responds to environmental stresses and serves as an important resource for genome-assisted improvement in cold and drought resistance of the *Corylus* genus.

Background

The *Corylus* genus, a member of the birch family Betulaceae and an economically and ecologically important nut tree species, is widely distributed throughout temperate regions of the Northern Hemisphere [1]. As a valuable nut crop, hazelnut provides the predominant flavor in a variety of cakes, candies, chocolate spreads, and butters. There is a high content of unsaturated fatty acids and several essential vitamins in hazelnut oil.

The number of *Corylus* species recognized by taxonomists ranges from 7 to 25, depending on different morphological and molecular classifications [2, 3]. Among these, the European hazelnut, *Corylus avellana* L., is the most widely commercially cultivated species, with more than 400 cultivars having been described [4]. Commercial cultivation of *C. avellana* is limited

to regions with climates moderated by large bodies of water that have cool summers and mild, humid winters, such as the slopes on the Black Sea of Turkey or the Willamette Valley of Oregon [5, 6]. Inadequate cold hardiness is a major factor limiting the expansion of commercial production into northern and inland areas. When *C. avellana* was introduced into China, twigs withered and died almost every winter due to the cold, windy, and dry climate in northern China. In southern China, however, European hazelnut trees seemed to grow well but bore few nuts, and abortive kernels were observed frequently [7].

Eight species and two botanical varieties of *Corylus* are reported to be native to China [5]. The Asian hazel *Corylus heterophylla* (NCBI:txid80754) is one of the most important economic *Corylus* species. Among the 1.67 million ha of wild *Corylus* in China, *C. heterophylla* occupies 90% of the geographic area [8]. Wild *C. heterophylla* is mainly distributed in the mountains from northern to northeastern China. The geographical distribution range is 36.78–51.73 (°N) and 100.57–132.20 (°E), where the main climate type is temperate. Compared with *C. avellana*, *C. heterophylla* can be adapted to regions with low temperatures (–30 to –40 °C) and drought conditions. Therefore, the cold and drought resistance characteristics of *C. heterophylla* can be used as parent materials for cross-breeding with other hazel species.

In the present study, to better understand the molecular mechanism of how hazelnuts respond to environmental stress, we assembled a high-quality genome of *C. heterophylla* using a combination of the Oxford Nanopore high-throughput sequencing technology and the high-throughput chromosome conformation capture (Hi-C) technique. Long reads were *de novo* assembled into 1,328 polished contigs with a total size of 370.75 Mb and contig N50 and scaffold N50 values of 2.07 Mb and 31.33 Mb, respectively, which is in line with genome sizes estimated using flow cytometry and *k*-mer analysis. A total of 361.90 Mb contigs were anchored into 11 chromosomes, representing 97.61% of the assembled genome. Our results provide a high-quality, chromosome-level genome assembly of *C. heterophylla*, which will support breeding programs leading to genetic improvement of hazelnuts. Furthermore, it will facilitate understanding of the special position of *Corylus* and Betulaceae in plant evolution.

Data Description

Sample collection

Fresh and healthy leaves were collected from a single wild *C. heterophylla* tree in Yanqing, Beijing, China (N: 40° 32′ 27″; E: 116° 03′ 52″; Fig. 1). The fresh leaf tissue was flash-frozen in liquid nitrogen for 30 min and then stored at –80 °C. DNA was extracted from leaf tissues following a previously published protocol [9]. Different tissues, including root, stem, staminate inflorescence, and leaf, were sampled and flash-frozen in liquid nitrogen for total RNA sequencing. Total RNA was extracted using the modified CTAB method [10].

Library preparation and whole-genome sequencing

Genomic DNA for library construction was isolated from leaf tissues using the DNeasy Plant Mini Kit (Qiagen, Beijing, CHN) according to the manufacturer’s instructions. DNA concentrations and quality were measured using a NanoDrop 2000 (Thermo Fisher, Waltham, MA, USA) and Qubit Fluorometer (Thermo Fisher, Waltham, MA, USA), respectively. The gDNA was sheared to ~500 bp fragments using an S2 Focused-Ultrasonicator (Covaris Inc., Woburn, MA, USA). Paired-end (PE) libraries were prepared using the TruSeq DNA PCR-Free Library Preparation Kit (Illumina, San Diego, CA, USA) according to the Illumina standard protocol. After quality control by an Agilent 2100 Bioanalyzer and qPCR, all PCR-free libraries were sequenced on an Illumina HiSeq X Ten system (Illumina, San Diego, CA, USA; RRID:SCR_016385) with a 350 bp PE sequencing strategy according to the manufacturer’s instructions. A total of 38.02 Gb (~102.55-fold coverage) clean reads were generated for the genome survey and Nanopore genome polishing (Supplementary Table S1a).

Estimation of genome size and heterozygosity analysis

Before genome assembly, we estimated the *C. heterophylla* genome’s size using Jellyfish (RRID:SCR_005491) [11] with an optimal *k*-mer size. A total of 38.02 Gb short reads (~102.55 x) were processed by Jellyfish to assess their *k*-mer distribution (*k*-mer value = 19). Theoretically, the *k*-mer frequency follows a Poisson distribution. We selected $k = 19$ for the genome size estimation in this study. Genome sizes were calculated from the following equation: Genome size = 19-mer number / 19-mer depth, where 19-mer number is the total counts of each unique 19-mer and 19-mer depth is the highest frequency that occurred (Supplementary Fig. S1). The estimated genome size of *C. heterophylla* is 373.61 Mb.

Nanopore, RNA, and Hi-C sequencing

Genomic DNA was extracted and sequenced following the instructions of the Ligation Sequencing Kit (Oxford Nanopore Technologies, Oxford, UK). DNA quality was assessed by agarose gel electrophoresis and NanoDrop 2000c spectrophotometry, followed by Thermo Fisher Scientific Qubit fluorometry. After quality control, genomic DNA was size-selected using a Blue Pippin BLF7510 cassette (Sage Science, Beverly, MA, USA). Libraries (fragments > 20 kb) were prepared using the standard Ligation Sequencing kit (SQK-LSK109; Oxford Nanopore Technologies, Oxford, UK) and sequenced on the GridION X5 platform (Oxford Nanopore Technologies, Oxford, UK) with FLOMIN106 (R9.4) flow cells. Raw ONT reads (fastq) were extracted from base-called FAST5 files using poretools [12]. Then, the short reads (<5 kb) and reads having low-quality bases and adapter sequences (YSFRI, 2019c) were removed. A total of 64.99 Gb (~175.30-fold coverage) Nanopore long reads with an N50 length of 27.17 kb were produced for genome assembly (Supplementary Fig. S2, Supplementary Tables S1b and S1c).

Different tissues, including leaf, stem, root, and staminate inflorescence, were harvested and flash-frozen in liquid nitrogen for total RNA sequencing. Each sample was subjected to poly(A) purification using oligo-dT beads (Thermo Fisher, Waltham, MA, USA) followed by ribosomal (rRNA) removal using the Ribo-Zero rRNA Removal Kit (Illumina，San Diego, CA, USA). The RNA quality was measured by 2100 RNA Nano 6000 Assay Kit (Agilent Technologies, Santa Clara, CA, USA) and pooling together. The resulting RNA sample was used for cDNA library construction using the NEBNext Ultra RNA Library Prep Kit for Illumina (NEB, Ipswich, MA, USA). The quantified libraries were then prepared for sequencing on the Illumina HiSeq X Ten system, producing 9.66 Gb PE reads (Supplementary Table S1d).

Hi-C experiments were performed as described with some modifications [13, 14]. Briefly, 2 g of freshly harvested leaves were cut into 2- to 3-mm pieces and infiltrated in 2% formaldehyde before cross-linking was stopped by adding glycine. The tissue was ground to powder and suspended in nuclei isolation buffer to obtain a nuclei suspension. The procedure for the Hi-C experiment, including chromatin digestion, labeling of DNA ends, DNA ligation, purification, and fragmentation, was performed as described previously [15]. The cross-linked DNA was

digested with HindIII as previously described and marked by incubating with Klenow enzyme and biotin-14-dCTP overnight at 37 °C [15]. The 5' overhang of the fragments was repaired and labeled using biotinylated nucleotides, followed by ligation with T4 DNA polymerase. After reversal of cross-linking, ligated DNA was purified and sheared to 300–700 bp fragments using an S2 Focused-Ultrasonicator (Covaris Inc., MA, USA). The linked DNA fragments were enriched with streptavidin beads and prepared for Illumina HiSeq X Ten sequencing, producing 231.31 Mb (totaling ~69.11 Gb) Hi-C links data (Supplementary Table S1e).

De novo genome assembly and pseudo-chromosome construction

After the self-error correction using the error correction model in Canu (Canu, RRID: SCR_015880) v1.5 [16], the Nanopore long reads were assembled into contigs using WTDBG2 (WTDBG, RRID: SCR_017225) v1.0 [17]. Two rounds of consensus correction were performed using Racon (Racon, RRID: SCR_017642) v1.32 [18] with corrected Nanopore long reads, and the resulting assembly was further polished using Pilon (Pilon, RRID: SCR_014731) [19] with 38.02 Gb Illumina short reads (Supplementary Table S1a). The assembled length of 1,291 contigs of *C. heterophylla* is 370.71 Mb, accounting for 99.22% of the estimated genome size (373.61 Mb). The contigs N50 and N90 were 2.11 Mb and 138.6 kb, respectively.

The pseudo-chromosomes were constructed using Hi-C links data. The clean Hi-C reads were mapped to the consensus contigs using the Burrows-Wheeler Aligner [20] (BWA, RRID: SCR 010910) v0.7.17, and only uniquely mapped read pairs were considered as high-quality read pairs in Hi-C analysis. The reads were removed if the mapped positions in the reference genome were further than 500 bp from the nearest restriction enzyme site. The quality assessment and normalization were performed using HiC-Pro (HiC-Pro, RRID: SCR_017643) [21]. There were 109,306,012 uniquely mapped PE reads, of which 58.33% (63,755,940) uniquely mapped reads were considered valid interaction pairs for chromosome construction (Supplementary Table S2). The contigs were then clustered, ordered, and oriented into 11 pseudo-chromosomes using LACHESIS (LACHESIS, RRID: SCR_017644) [21]. Finally, we obtained a high-quality chromosome-level reference genome with a total size of 370.75 Mb. The contig N50 and scaffold N50 values were 2.07 Mb and 31.33 Mb, respectively (Table 1). A total of 361.90 Mb

contigs were anchored into 11 chromosomes, representing 97.61% of the assembled genome (Table 2).

Genome quality assessment

Genome completeness was assessed using the plants dataset of the Benchmarking Universal Single-Copy Orthologs (BUSCO, RRID: SCR_015008) database v1.22 [22], with e-value < $1e^{-5}$. The BUSCO database detected 93.47% and 1.18% of complete and partial gene models, respectively, in the *C. heterophylla* assembly results (Table 3). The core eukaryotic gene-mapping approach (CEGMA, RRID: SCR_015055) [23] provides a method to rapidly assess genome completeness because it comprises a set of highly conserved, single-copy genes, present in all eukaryotes, containing 458 core eukaryotic genes (CEGs). We identified CEGs using the CEGMA (CEGMA, RRID: SCR_015055) v2.3 pipeline [23] and found that 430 (93.89%) CEGs could be found in the assembly results (Supplementary Table S3a). The PE short libraries, including 103,392,992 paired reads, were remapped to the assembly genome with BWA-MEM (BWA, RRID: SCR 010910) [24] to assess the completeness of the assembly results. More than 98.47% of these reads could be accurately mapped into genome sequences (Supplementary Table S3b). Additionally, the heatmap of the Hi-C interaction frequency was selected to visually assess the assembled accuracy of the *C. heterophylla* genome. The interaction heatmap was displayed at 100 kb resolution. LG01-LG11 represent the eleven chromosomes of the *C. heterophylla* genome ordered by chromosome length. The horizontal and vertical coordinates represent the order of each ‘bin’ on the corresponding chromosome. The signal intensities clearly divide the ‘bins’ into eleven distinct groups (LG01-LG11), demonstrating the high quality of the chromosome assignment (Fig. 2). These observations suggest the high quality and completeness of this chromosome-level reference genome for *C. heterophylla*.

Repetitive elements and protein-coding gene annotation

Repetitive elements in the *C. heterophylla* genome were identified using a combined strategy of *de novo* and homology-based approaches at the DNA and protein levels. Tandem repeats were annotated using Tandem Repeat Finder (TRF). A repeat library was constructed using

MITE-Hunter (MITE-Hunter RRID: SCR_020946) [25], LTR-FINDER (LTR Finder, RRID: SCR_015247) v1.05 [26], RepeatScout (RepeatScout, RRID: SCR_014653) v1.0.5 [27], and PILER (PILER, RRID: SCR_017333) [28] for *de novo* repeat content annotation. The *de novo* repeat library was classified through PASTEClassifier (PASTEClassifier, RRID: SCR_017645) v1.0 package [29] with default parameters and then integrated with Repbase（Repbase, RRID: SCR_012954） v19.06 [30] to build a new repeat library. Finally, RepeatMasker (RepeatMasker, RRID: SCR_012954) v4.0.6 [31] with parameters of "-nolow -no_is -norna -engine wublast" was selected to identify and classify the genomic repetitive elements of *C. heterophylla.* In total, 210.26 Mb of repetitive sequences were identified, accounting for 56.71% of *C. heterophylla* genome sequences (Table 4). The top three classes of repetitive elements were ClassI/LARD, ClassI/LTR/Gypsy, and ClassI/LTR/Copia, occupying 20.51%, 11.14%, and 10.44% of assembled genome sequences, respectively (Table 4).

Gene annotation was performed using a combination of *ab initio* prediction, homology-based gene prediction, and transcript evidence from RNA-seq data. The *de novo* approach was implemented using Augustus (Augustus, RRID: SCR_008417) v3.2.3 [32], GeneID (GeneID, RRID: SCR_002473) v1.4.4 [33], GlimmerHMM (GlimmerHMM, RRID: SCR_002654) v3.52 [34], GenScan (GENSCAN, RRID: SCR_012902) [35], and SNAP (SNAP, RRID: SCR_007936) [36]. For homology-based prediction, TBLASTN (TBLASTN, RRID: SCR_011822) v2.2.31 [37] was used to align predicted protein sequences of *Arabidopsis thaliana*, *Betula pendula*, *Juglans regia* and *Ostrya chinensis* to the *C. heterophylla* genome with an e-value threshold of $1e^{-5}$. Then, GeMoMa (GeMoMa, RRID: SCR_017646) v1.3.1 [38] was employed for homology-based gene prediction. The transcriptome data from pooled tissues of leaf, stem, root, and staminate inflorescence from *C. heterophylla* were assembled into unigenes using HISAT (HISAT, RRID: SCR_015530) v2.0.4 [39] and StringTie (StringTie, RRID: SCR_016323) v1.2.3 [40]. Then unigenes were used to predict gene structures using TransDecoder (TransDecoder, RRID: SCR_017647) v2.0 [41], GeneMarkS-T (GeneMarkS-T, RRID: SCR_017648) v5.1 [42], and PASA (PASA, RRID: SCR_014656) v2.0.2 [43]. Finally, the gene models obtained from the above three approaches were integrated into a consensus gene set using EVidenceModeler (EVidenceModeler, RRID: SCR_014659) v1.1.0 [44] with default parameters. PASA (PASA, RRID: SCR_014656) v2.0.2 [43] was then used to annotate

the gene structures, including UTRs and alternative-splice sites (Supplementary Fig. S3, Supplementary Table S4a). A total of 27,591 non-redundant protein-coding genes were predicted for the *C. heterophylla* genome (Table 1). Gene models were annotated by homologous searching against several databases using BLASTP (BLASTP, RRID: SCR_001010) from BLAST+ package [37] (e-value = 1e^{-5}), including NR [45], KOG [46], TrEMBL (TrEMBL, RRID: SCR_002380) [47], and KEGG (KEGG, RRID: SCR_012773) [48]databases. InterProScan (InterProScan, RRID: SCR_005829) v4.3 [49] was used to annotate the protein motifs and domains. The Blast2GO (Blast2GO, RRID: SCR_005828) [50, 51] pipeline was used to obtain GO terms annotation from the NCBI NR database. In total, 25,389 protein-coding genes (92.02%) were successfully assigned into corresponding functions (Supplementary Table S4b).

Genome-wide pseudogene identification was carried out for *C. heterophylla*. Only candidate pseudogenes containing frameshifts and/or premature stop codons in their coding regions were considered as reliable pseudogenes. *C. heterophylla* proteins were aligned to the reference genome using GenBlastA (GenBlastA, RRID:SCR_020951) v1.0.4 [52] to detect candidate homolog regions. Then, the candidate pseudogenes were identified using GeneWise (GeneWise, RRID: SCR_015054) v2.4.1 [53]. Finally, 2,988 pseudogenes were identified in *C. heterophylla* genome sequences (Table 1).

Different types of non-coding RNA in the *C. heterophylla* genome were identified and classified as family and subfamily. The tRNAscan-SE (tRNAscan-SE, RRID: SCR_010835) v1.23 [54] was applied to detect transfer RNAs (tRNAs). MicroRNAs (miRNAs) were identified by homolog searching miRBase (microRNA database (miRBase), RRID: SCR_003152) v21 [55] against the *C. heterophylla* genome with one mismatch. Then, secondary structures of the putative sequences were predicted by miRDeep2 (miRDeep, RRID: SCR_010829) [56]. Finally, putative miRNAs with hairpin structures were considered as reliable ones. Other types of non-coding RNA were detected using Infernal (Infernal, RRID: SCR_011809) [57] (e-value ≤ 0.01) based on the Rfam database (Rfam, RRID: SCR_007891) v12.0 [58]. In total, 92 miRNAs, 617 tRNAs, and 622 rRNAs were annotated in *C. heterophylla* genome sequences (Supplementary Table S4c).

Gene family identification and phylogenetic tree construction

In the gene family and phylogenetic analysis, the protein-coding genes of *Oryza sativa*, *Arabidopsis thaliana, Populus trichocarpa, Quercus variabilis, Juglans regia, Betula pendula*, *Ostrya japonica*, and *C. heterophylla* were downloaded from Genbank or Ensembl databases. The longest transcripts were selected to represent the protein-coding genes. Protein sequence clustering was performed using OrthoMCL (OrthoMCL, RRID: SCR_007839) v2.0 [59] with default parameters to identify the orthologous groups. The result showed that *C. heterophylla* has 16,811 orthologous groups, including 5,150 single-copy genes, 6,040 multiple-copy genes, and 582 specific genes. Notably, 222 species-specific families were identified for *C. heterophylla*, which might contribute to its unique features (Fig. 3A). To construct the phylogenetic analysis, 1,182 single-copy orthologs were identified from one copy families of selected species. The protein sequences of single-copy orthologs were aligned using MUSCLE (MUSCLE, RRID: SCR_011812) v3.8.31 [60], and low-quality alignment regions were removed using Gblocks (Gblocks, RRID: SCR_015945) v0.91b [61] with default parameters. A phylogenetic tree was constructed using the maximum-likelihood method with the JTT amino acid substitution model implemented in the PhyML (PhyML, RRID: SCR_014629) v3.3 package [62]. The divergence time was estimated using the MCMCtree program in the PAML (Phylogenetic Analysis of Maximum-Likelihood; PAML, RRID: SCR_014932) v4.7b package [63]. An age of (51.2 - 66.7 Mya) was used to calibrate the crown nodes of the family Betulaceae [64]. The monocot-dicot split time (152 - 160 Mya) obtained from the TimeTree database was also used to calibrate the time estimation [65]. The result showed that *C. heterophylla* is close to *O. japonica*, and they diverged from their common ancestor ∼52.79 million years ago (Fig. 3B).

Conclusion

To our knowledge, this is the first report of a chromosome-level genome assembly of *C. heterophylla* using the third-generation sequencing technologies of Nanopore and Hi-C. *C. heterophylla* has 210.26 Mb of repetitive sequences, accounting for 56.71% of genomic sequences. A total of 25,389 high-quality protein-coding genes were annotated by integrating evidence from *de novo* prediction, homologous protein prediction, and transcriptome data.

Phylogenetic analysis showed that *Corylus* is closely related to *Ostrya*, and they diverged from their common ancestor approximately 52.79 Mya. This work provides valuable chromosome-level genomic data for studying loquat traits. The genomic data should promote research on the molecular mechanisms of hazelnut responses to environmental stress and provides a valuable resource for genome-assisted improvements in *Corylus* breeding.

Additional Files

Supplementary Figure S1: Genome survey analysis of *C. heterophylla* based on *k*-mer = 19.

Supplementary Figure S2: Fragment size distribution of Hi-C read pairs.

Supplementary Figure S3: Venn plot of predicted genes generated from *ab initio*, RNAseq, and homology methods.

Supplementary Table S1a: Summary of Illumina data for genome survey and genome polishing.

Supplementary Table S1b: Statistics of Nanopore long reads.

Supplementary Table S1c: Distribution of Nanopore long read lengths.

Supplementary Table S1d: Summary of pooled transcriptome data used for gene prediction.

Supplementary Table S1e: Summary of Hi-C data for error correction and chromosome construction.

Supplementary Table S2: Valid interaction pairs of Hi-C sequencing data.

Supplementary Table S3a: Completeness analysis based on the CEG database.

Supplementary Table S3b: Genome completeness assessment based on Illumina sequencing reads.

Supplementary Table S4a: Summary of gene predictions resulting from different evidence.

Supplementary Table S4b: Gene function annotated by different databases.

Supplementary Table S4c: Non-coding RNA identification.

Abbreviations

BLAST: Basic Local Alignment Search Tool; bp: base pairs; BUSCO: Benchmarking Universal Single-Copy Orthologs; BWA: Burrows-Wheeler Aligner; CEGMA: Core Eukaryotic Genes Mapping Approach; CTAB: Hexadecyltrimethylammonium Bromide; Gb: gigabase pairs; GeMoMa: Gene Model Mapper; GO: Gene Ontology; Hi-C: high-throughput chromosome

conformation capture; HiSeq: high-throughput sequencing; HMM: hidden Markov model; kb: kilobase pairs; KEGG: Kyoto Encyclopedia of Genes and Genomes; KOG: EuKaryotic Orthologous Groups; LG: linkage group; LTR: long terminal repeat; Mb: megabase pairs; miRNA: microRNA; MITE: miniature inverted-repeat transposable element; MUSCLE: MUltiple Sequence Comparison by Log-Expectation; Mya: million years ago; NCBI: National Center for Biotechnology Information; NR: non-redundant; PAML: Phylogenetic Analysis of Maximum-Likelihood; PASA: Program to Assemble Spliced Alignments; PCR: polymerase chain reaction; PE: paired-end; PhyML: Phylogeny Maximum Likelihood; RNA-seq: RNA sequencing; rRNA: ribosomal RNA; SAAS: Shanghai Academy of Agricultural Sciences; SNAP: Semi-HMM-based Nucleic Acid Parser; TIR: terminal inverted repeat; TrEMBL: a database of translated proteins from European Bioinformatics Institute; TRF: Tandem Repeat Finder; tRNA: transfer RNA.

Competing Interests

The authors declare that they have no competing interests.

Funding

This work was financed by the Special Investigation on Basic Resources of Science and Technology (Grant No. 2019FY100801_03) and the Special Fund for Basic Scientific Research Business of Central Public Research Institutes of the Chinese Academy of Forestry, China (Grant No. RIF-12 and CAFYBB2017ZA004-9).

Authors' Contributions

T.Z., Z.Y., W.M., Q.M., and L.W. designed and conceived the study; W.M., L.L., and G.X. helped to collect the samples; T.Z., Z.Y., L.L., Q.M., and L.W. performed the experiments; T.Z., W.M., Z.Y., Q.M., X.C., and L.W. wrote and revised the manuscript. All authors read and approved the manuscript.

Availability of supporting data

The genome sequence data have been deposited in NCBI under the accession

JADOBO000000000. Raw reads of Nanopore, WGS, Hi-C and RNAseq, and genome assembly sequences of the *C. heterophylla* genome have been deposited at the Nucleotide Sequence Archive and GenBank in NCBI under BioProject PRJNA655406 and BioSample Accessions of SAMN15734705 and SAMN15734794. The SRA Accessions are SRR12458330, SRR12458329, SRR12458328, SRR12458327. All supplementary figures and tables are provided in Additional Files. Additional supporting data, including annotations, RNA-seq data, and phylogenetic trees, are available in the GigaDB database [66].

Acknowledgements

We thank Changmian Ji from the Chinese Academy of Tropical Agricultural Sciences for assistance with bioinformatics analysis and discussion.

References

1. Zong JW, Zhao TT, Ma QH, et al. Assessment of genetic diversity and population genetic structure of *Corylus mandshurica* in china using SSR markers. PLoS One. 2015;10 9:e0137528. doi:10.1371/journal.pone.0137528.
2. Mehlenbacher SA. Hazelnuts. A guide to nut tree culture in north america. Northern Nut Growers Association, Inc; 2003.
3. Boccacci P, Beltramo C, Sandoval Prando MA, et al. In silico mining, characterization and cross-species transferability of EST-SSR markers for European hazelnut (*Corylus avellana* L.). Molecular Breeding. 2015;35 1:21. doi:10.1007/s11032-015-0195-7.
4. Gürcan K, Mehlenbacher S, Botta R, et al. Development, characterization, segregation, and mapping of microsatellite markers for European hazelnut (*Corylus avellana* L.) from enriched genomic libraries and usefulness in genetic diversity studies. Tree Genetics & Genomes. 2010;6 4:513-31.
5. Zhang YH, Liu L, Liang WJ, et al. China fruit's monograph-chestnut and hazelnut. Beijing:China Forestry Publishing House; 2005.
6. Molnar TJ. *Corylus*. Wild crop relatives: Genomic and breeding resources. 1 ed. Forest Trees: Springer-Verlag Berlin Heidelberg; 2011.
7. Wang GX. Studies on the cultivation and utilization of *Corylus* resources in china (Ⅰ) - *Corylus* germplasm resources. Forestry Science Research. 2018;31:105 -12.
8. Wang GX, Ma QH, Zhao TT, et al. Resources and production of hazelnut in china. Acta horticulturae. 2018; 1226:59-64.
9. Mayjonade B, Gouzy J, Donnadieu C, et al. Extraction of high-molecular-weight genomic DNA for long-read sequencing of single molecules. Biotechniques. 2016;61 4:203-5. doi:10.2144/000114460.
10. Doyle J. A rapid DNA isolation procedure for small quantities of fresh leaf tissue. Phytochem Bull. 1987;19.
11. Marçais G, Kingsford C. A fast, lock-free approach for efficient parallel counting of occurrences of *k*-mers. Bioinformatics. 2011;27 6:764-70. doi:10.1093/bioinformatics/btr011.

12. Loman NJ, Quinlan AR. Poretools: A toolkit for analyzing nanopore sequence data. Bioinformatics. 2014;30 23:3399-401. doi:10.1093/bioinformatics/btu555.

13. Belton JM, McCord RP, Gibcus JH, et al. Hi-C: A comprehensive technique to capture the conformation of genomes. Methods. 2012;58 3:268-76. doi:10.1016/j.ymeth.2012.05.001.

14. Grob S, Schmid MW, Grossniklaus U. Hi-C analysis in arabidopsis identifies the KNOT, a structure with similarities to the flamenco locus of *Drosophila*. Mol Cell. 2014;55 5:678-93. doi:10.1016/j.molcel.2014.07.009.

15. Xie T, Zheng JF, Liu S, et al. *De novo* plant genome assembly based on chromatin interactions: A case study of *Arabidopsis thaliana*. Mol Plant. 2015;8 3:489-92. doi:10.1016/j.molp.2014.12.015.

16. Koren S, Walenz BP, Berlin K, et al. Canu: Scalable and accurate long-read assembly via adaptive k-mer weighting and repeat separation. Genome Res. 2017;27 5:722-36. doi:10.1101/gr.215087.116.

17. Ruan J, Li H. Fast and accurate long-read assembly with wtdbg2. Nat Methods. 2020;17 2:155-8. doi:10.1038/s41592-019-0669-3.

18. Vaser R, Sović I, Nagarajan N, et al. Fast and accurate *de novo* genome assembly from long uncorrected reads. Genome Research. 2017;27 5:737-46. doi:10.1101/gr.214270.116.

19. Walker BJ, Abeel T, Shea T, et al. Pilon: An integrated tool for comprehensive microbial variant detection and genome assembly improvement. PLoS One. 2014;9 11:e112963. doi:10.1371/journal.pone.0112963.

20. Li H, Durbin R. Fast and accurate short read alignment with burrows-wheeler transform. Bioinformatics. 2009;25 14:1754-60. doi:10.1093/bioinformatics/btp324.

21. Burton JN, Adey A, Patwardhan RP, et al. Chromosome-scale scaffolding of *de novo* genome assemblies based on chromatin interactions. Nat Biotechnol. 2013;31 12:1119-25. doi:10.1038/nbt.2727.

22. Seppey M, Manni M, Zdobnov EM. BUSCO: Assessing genome assembly and annotation completeness. Methods Mol Biol. 2019;1962:227-45. doi:10.1007/978-1-4939-9173-0_14.

23. Parra G, Bradnam K, Korf I. CEGMA: A pipeline to accurately annotate core genes in eukaryotic genomes. Bioinformatics. 2007;23 9:1061-7. doi:10.1093/bioinformatics/btm071.

24. Li H, Durbin R. Fast and accurate long-read alignment with Burrows-Wheeler transform. Bioinformatics. 2010;26 5:589-95. doi:10.1093/bioinformatics/btp698.

25. Han Y, Wessler SR. MITE-Hunter: A program for discovering miniature inverted-repeat transposable elements from genomic sequences. Nucleic acids research. 2010;38 22:e199. doi:10.1093/nar/gkq862.

26. Xu Z, Wang H. LTR_FINDER: An efficient tool for the prediction of full-length LTR retrotransposons. Nucleic Acids Res. 2007;35 Web Server issue:W265-8. doi:10.1093/nar/gkm286.

27. Price AL, Jones NC, Pevzner PA. *De novo* identification of repeat families in large genomes. Bioinformatics. 2005;21 Suppl 1:i351-8. doi:10.1093/bioinformatics/bti1018.

28. Edgar RC, Myers EW. PILER: Identification and classification of genomic repeats. Bioinformatics. 2005;21 Suppl 1:i152-8. doi:10.1093/bioinformatics/bti1003.

29. Hoede C, Arnoux S, Moisset M, et al. PASTEC: An automatic transposable element classification tool. PLoS One. 2014;9 5:e91929. doi:10.1371/journal.pone.0091929.

30. Bao W, Kojima KK, Kohany O. Repbase update, a database of repetitive elements in eukaryotic genomes. Mob DNA. 2015;6:11. doi:10.1186/s13100-015-0041-9.

31. Tarailo-Graovac M, Chen N. Using RepeatMasker to identify repetitive elements in genomic sequences. Curr Protoc Bioinformatics. 2009;Chapter 4:Unit 4.10. doi:10.1002/0471250953.bi0410s25.

32. Stanke M, Morgenstern B. AUGUSTUS: A web server for gene prediction in eukaryotes that allows user-defined constraints. Nucleic acids research. 2005;33 Web Server issue:W465-7. doi:10.1093/nar/gki458.

33. Alioto T, Blanco E, Parra G, et al. Using geneid to identify genes. Curr Protoc Bioinformatics. 2018;64 1:e56. doi:10.1002/cpbi.56.
34. Majoros WH, Pertea M, Salzberg SL. TigrScan and GlimmerHMM: Two open source *ab initio* eukaryotic gene-finders. Bioinformatics. 2004;20 16:2878-9. doi:10.1093/bioinformatics/bth315.
35. Burge C, Karlin S. Prediction of complete gene structures in human genomic DNA. J Mol Biol. 1997;268 1:78-94. doi:10.1006/jmbi.1997.0951.
36. Korf I. Gene finding in novel genomes. BMC Bioinformatics. 2004;5:59. doi:10.1186/1471-2105-5-59.
37. Camacho C, Coulouris G, Avagyan V, et al. Blast+: Architecture and applications. BMC Bioinformatics. 2009;10:421. doi:10.1186/1471-2105-10-421.
38. Keilwagen J, Wenk M, Erickson JL, et al. Using intron position conservation for homology-based gene prediction. Nucleic acids research. 2016;44 9:e89. doi:10.1093/nar/gkw092.
39. Kim D, Langmead B, Salzberg SL. Hisat: A fast spliced aligner with low memory requirements. Nat Methods. 2015;12 4:357-60. doi:10.1038/nmeth.3317.
40. Pertea M, Pertea GM, Antonescu CM, et al. Stringtie enables improved reconstruction of a transcriptome from RNA-seq reads. Nat Biotechnol. 2015;33 3:290-5. doi:10.1038/nbt.3122.
41. TransDecoder (find coding regions within transcripts). Version 5.5.0. https://github.Com/transdecoder/transdecoder. Accessed 10 February 2020.
42. Tang S, Lomsadze A, Borodovsky M. Identification of protein coding regions in RNA transcripts. Nucleic acids research. 2015;43 12:e78. doi:10.1093/nar/gkv227.
43. Campbell MA, Haas BJ, Hamilton JP, et al. Comprehensive analysis of alternative splicing in rice and comparative analyses with arabidopsis. BMC Genomics. 2006;7:327. doi:10.1186/1471-2164-7-327.
44. Haas BJ, Salzberg SL, Zhu W, et al. Automated eukaryotic gene structure annotation using EVidenceModeler and the program to assemble spliced alignments. Genome Biol. 2008;9 1:R7. doi:10.1186/gb-2008-9-1-r7.
45. Deng YY, Li JQ, Wu SF, et al. Integrated NR database in protein annotation system and its localization. Computer Engineering. 2006;32 5:71-2.
46. Koonin EV, Fedorova ND, Jackson JD, et al. A comprehensive evolutionary classification of proteins encoded in complete eukaryotic genomes. Genome Biol. 2004;5 2:R7. doi:10.1186/gb-2004-5-2-r7.
47. Boeckmann B, Bairoch A, Apweiler R, et al. The SWISS-PROT protein knowledgebase and its supplement TrEMBL in 2003. Nucleic acids research. 2003;31 1:365-70. doi:10.1093/nar/gkg095.
48. Kanehisa M, Furumichi M, Tanabe M, et al. KEGG: New perspectives on genomes, pathways, diseases and drugs. Nucleic acids research. 2017;45 D1:D353-d61. doi:10.1093/nar/gkw1092.
49. Zdobnov EM, Apweiler R. InterProScan--an integration platform for the signature-recognition methods in interPro. Bioinformatics. 2001;17 9:847-8. doi:10.1093/bioinformatics/17.9.847.
50. Conesa A, Götz S. Blast2GO: A comprehensive suite for functional analysis in plant genomics. Int J Plant Genomics. 2008;2008:619832. doi:10.1155/2008/619832.
51. Götz S, García-Gómez JM, Terol J, et al. High-throughput functional annotation and data mining with the Blast2GO suite. Nucleic acids research. 2008;36 10:3420-35. doi:10.1093/nar/gkn176.
52. She R, Chu JS, Wang K, et al. GenBlastA: Enabling BLAST to identify homologous gene sequences. Genome Res. 2009;19 1:143-9. doi:10.1101/gr.082081.108.
53. Birney E, Durbin R. Using GeneWise in the *Drosophila* annotation experiment. Genome Res. 2000;10 4:547-8. doi:10.1101/gr.10.4.547.
54. Lowe TM, Eddy SR. tRNAscan-SE: A program for improved detection of transfer RNA genes in genomic sequence. Nucleic acids research. 1997;25 5:955-64. doi:10.1093/nar/25.5.955.

55. Kozomara A, Birgaoanu M, Griffiths-Jones S. miRBase: From microRNA sequences to function. Nucleic acids research. 2019;47 D1:D155-d62. doi:10.1093/nar/gky1141.
56. Friedländer MR, Mackowiak SD, Li N, et al. miRDeep2 accurately identifies known and hundreds of novel microRNA genes in seven animal clades. Nucleic acids research. 2012;40 1:37-52. doi:10.1093/nar/gkr688.
57. Nawrocki EP, Eddy SR. Infernal 1.1: 100-fold faster rna homology searches. Bioinformatics. 2013;29 22:2933-5. doi:10.1093/bioinformatics/btt509.
58. Nawrocki EP, Burge SW, Bateman A, et al. Rfam 12.0: Updates to the RNA families database. Nucleic acids research. 2015;43 Database issue:D130-7. doi:10.1093/nar/gku1063.
59. Li L, Stoeckert CJ, Jr., Roos DS. OrthoMCL: Identification of ortholog groups for eukaryotic genomes. Genome Res. 2003;13 9:2178-89. doi:10.1101/gr.1224503.
60. Edgar RC. MUSCLE: Multiple sequence alignment with high accuracy and high throughput. Nucleic acids research. 2004;32 5:1792-7. doi:10.1093/nar/gkh340.
61. Talavera G, Castresana J. Improvement of phylogenies after removing divergent and ambiguously aligned blocks from protein sequence alignments. Syst Biol. 2007;56 4:564-77. doi:10.1080/10635150701472164.
62. Guindon S, Dufayard JF, Lefort V, et al. New algorithms and methods to estimate maximum-likelihood phylogenies: Assessing the performance of phyml 3.0. Syst Biol. 2010;59 3:307-21. doi:10.1093/sysbio/syq010.
63. Yang Z. PAML 4: Phylogenetic analysis by maximum likelihood. Mol Biol Evol. 2007;24 8:1586-91. doi:10.1093/molbev/msm088.
64. Takhtajan AL. Outline of the classification of flowering plants (*Magnoliophyta*). Botanical Review. 1980;46 3:225-359.
65. Timetree database. http://www.Timetree.Org/. Accessed 10 February 2020.
66. Zhao T, Ma W, Yang Z, et al. Supporting data for "A chromosome-level reference genome of the hazelnut, Corylus heterophylla Fisch." GigaScience Database 2021. http://doi.org/10.5524/100877

figures and tables

Click here to access/download;Figure;Figures and Tables-2021.2.19.docx

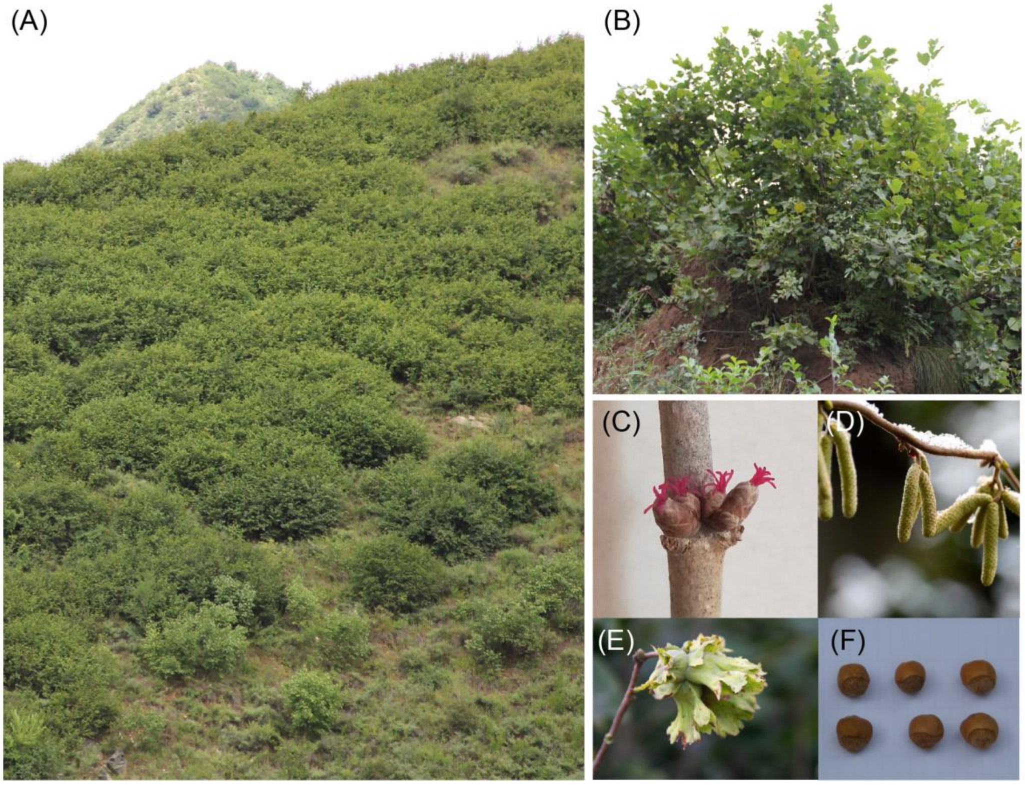

Figure1: Morphological characters of the Asian hazelnut variety, *C. heterophylla*. Mature plants in panel (A) and (B), female inflorescence (C), staminate inflorescence (D), fruit with husk (E), and nuts (F) are shown.

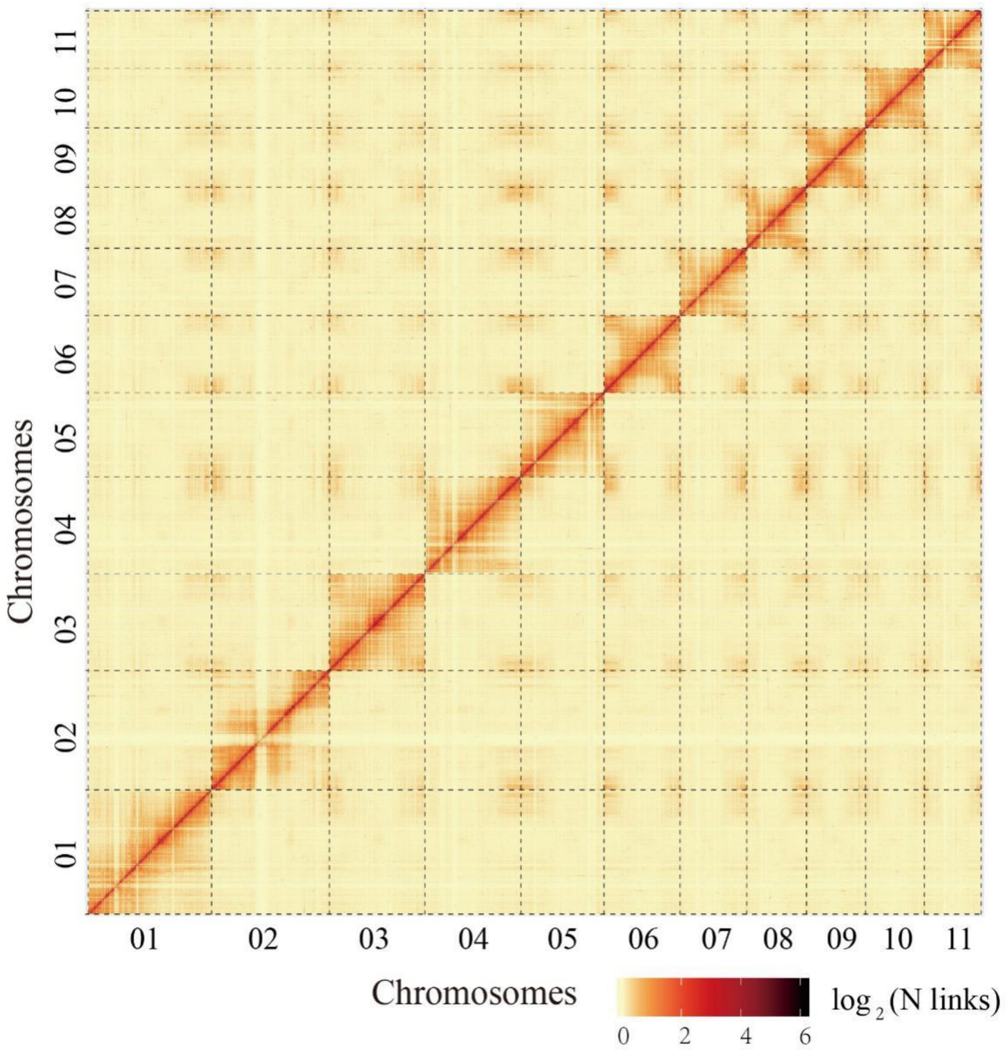

Figure2: Interaction frequency distribution of Hi-C links among eleven chromosomes. Genome-wide Hi-C map of *C. heterophylla*. We scanned the genome by 500-kb nonoverlapping window as a bin and calculated valid interaction links of Hi-C data between any pair of bins. The log2 of link number was transformed. The color key of heatmap ranging from light yellow to dark red represented the frequency of Hi-C interaction links from low to high (0~6).

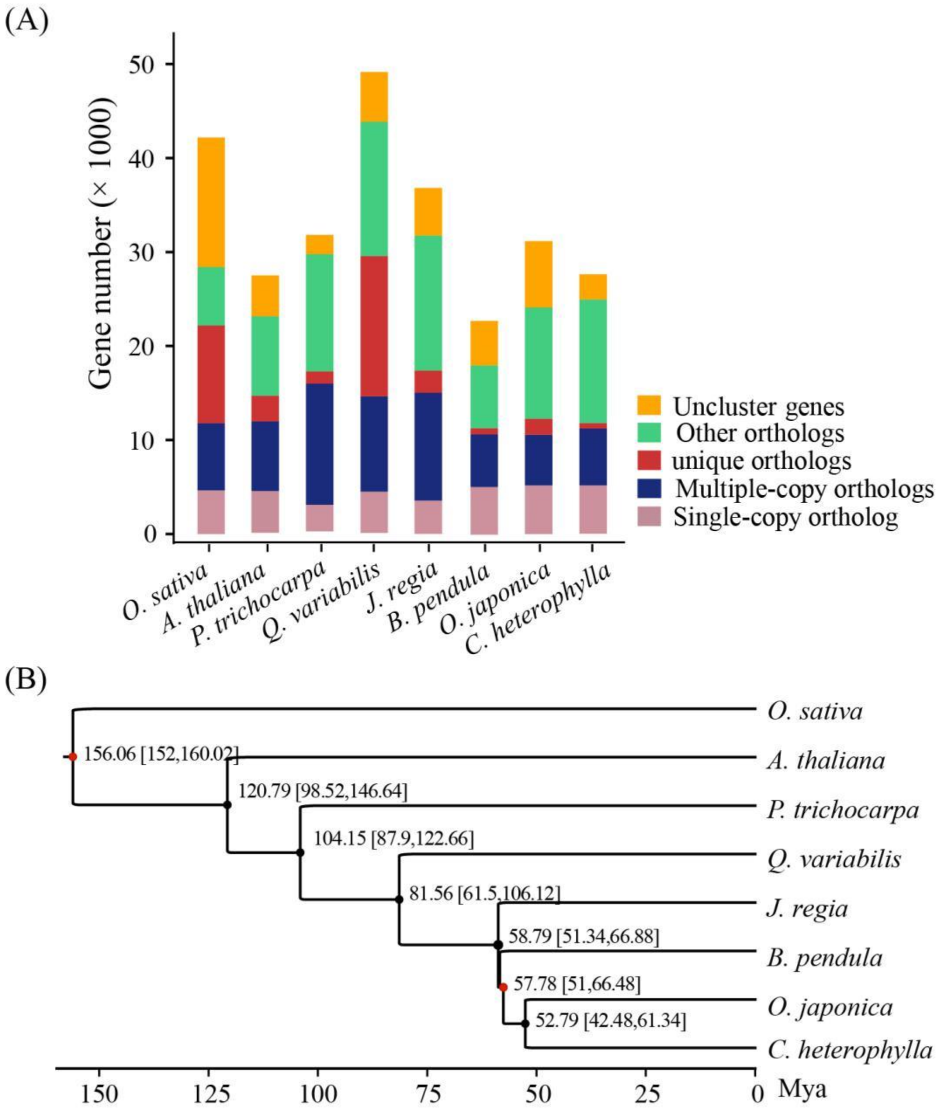

Figure3: Genome evolution analysis of *C. heterophylla*. (A) Summary of gene family clustering of *C. heterophylla* and 7 related species. Single-copy ortholog, one copy genes in ortholog group. Multiple-copy orthologs, multiple genes in ortholog group. Unique orthologs, species-specific genes. Other orthologs, the rest of the clustered genes. Uncluster genes, number of genes out of cluster. (B) Phylogenetic relationship and divergence time estimation (MYA, millions of years ago). The *O. sativa* was considered as outgroup in phylogenetic tree construction. The red dots indicate the fossil correction time of *O. sativa* vs *P. trichocarpa* (152 - 160 Mya) and crown nodes of family Betulaceae (51.2 - 66.7 Mya), respectively.

Table 1. Statistics of assembly results of *C. heterophylla* genome.

Feature	*C. heterophylla*
Genome size (bp)	370,750,808
Contig number	1,328
Maximum contig length (bp)	9,680,353
Contig N50 (bp)	2,068,510
Contig L50	48
Contig N90 (bp)	125,301
Scaffold number	951
Maximum scaffold length (bp)	46,514,939
Scaffold N50 (bp)	31,328,411
Scaffold L50	5
Scaffold N90 (bp)	21,561,575
GC content (%)	35.84
Gene number	27,591
Gene length (bp)	123,431,253
Average gene length (bp)	4,473.61
Exon number	138,886
Exon length (bp)	33,679,425
Intron number	138,885
Intron length (bp)	89,751,828
Pseudogenes	2,988
Pseudogene length (bp)	7,166,319

Note: only sequences whose length is more than 1 kb are considered.

Table 2. Summary of eleven pseudo-chromosomes for *C. heterophylla*.

Chr	No. of clustered sequences	Length of clustered sequences (bp)	No. of ordered sequences	Length of ordered sequences (bp)
LG01	114	49,577,893	56	46,509,439
LG02	113	48,019,691	49	44,425,769
LG03	67	37,395,073	33	36,016,943
LG04	95	38,562,170	53	36,392,613
LG05	85	34,656,877	37	31,324,811
LG06	76	31,263,564	31	28,814,739
LG07	103	29,494,057	36	25,003,895
LG08	45	23,716,498	23	22,749,571
LG09	41	23,427,462	17	22,292,654
LG10	41	23,093,417	25	22,249,747
LG11	53	22,694,573	28	21,558,875
Total (%)	833 (62.73)	361,901,275 (97.61)	388 (46.58)	337,339,056 (93.21)

Table 3. Genome completeness assessment by BUSCO.

Categories	Number	Percent (%)
Complete BUSCOs	1,346	93.47
Complete and single-copy BUSCOs	1,296	90.00
Complete and duplicated BUSCOs	50	3.47
Fragmented BUSCOs	17	1.18
Missing BUSCOs	77	5.35
Total BUSCO groups searched	1,440	100.00

Table 4. Repetitive elements in the *C. heterophylla* genome.

Classes	Number	Length (bp)	Percent (%)
ClassI	584,311	169,738,018	45.78
ClassI/DIRS	18,638	7,059,337	1.9
ClassI/LARD	303,288	76,033,830	20.51
ClassI/LINE	60,182	18,890,786	5.1
ClassI/LTR/Copia	101,158	38,719,023	10.44
ClassI/LTR/Gypsy	83,300	41,302,761	11.14
ClassI/LTR/Unknown	1,953	1,080,718	0.29
ClassI/PLE	5,600	4,125,513	1.11
ClassI/SINE	5,344	1,058,985	0.29
ClassI/TRIM	3,828	1,023,113	0.28
ClassI/Unknown	1,020	244,561	0.07
ClassII	77,407	24,382,510	6.58
ClassII/Crypton	455	109,226	0.03
ClassII/Helitron	27,254	8,348,317	2.25
ClassII/MITE	1,112	194,088	0.05
ClassII/Maverick	754	165,986	0.04
ClassII/TIR	44,403	15,342,483	4.14
ClassII/Unknown	3,429	459,116	0.12
PotentialHostGene	46,369	9,994,181	2.7
SSR	1,135	265,113	0.07
Unknown	116,728	26,584,597	7.17
Total	825,950	210,255,221	56.71

DIRS: dictyostelium intermediate repeat sequence; LARD: large retrotransposon derivative; LINE: long interspersed nuclear element; LTR: long terminal repeat; MITE: miniature inverted-repeat transposable element; PLE: Penelope-like element; SINE: short interspersed nuclear element; SSR: simple sequence repeat; TIR: terminal inverted repeat; TRIM: terminal-repeat retrotransposons in miniature.

Supplementary Tables

Click here to access/download
Supplementary Material
Supplementary tables.xls

Supplementary Figures

Click here to access/download
Supplementary Material
Supplementary figures.docx
