# Supplementary material for: A chromosome-level reference genome of the hazelnut, Corylus heterophylla Fisch
Source: Gigascience. 2021 Apr 19;10(4):giab027. doi: 10.1093/gigascience/giab027 (PMC8054262; doi:10.1093/gigascience/giab027)
Supplement: giab027_Supplemental_Figures_and_Tables [file giab027_supplemental_figures_and_tables.zip › Supplementary figures.docx]

Supplementary Material


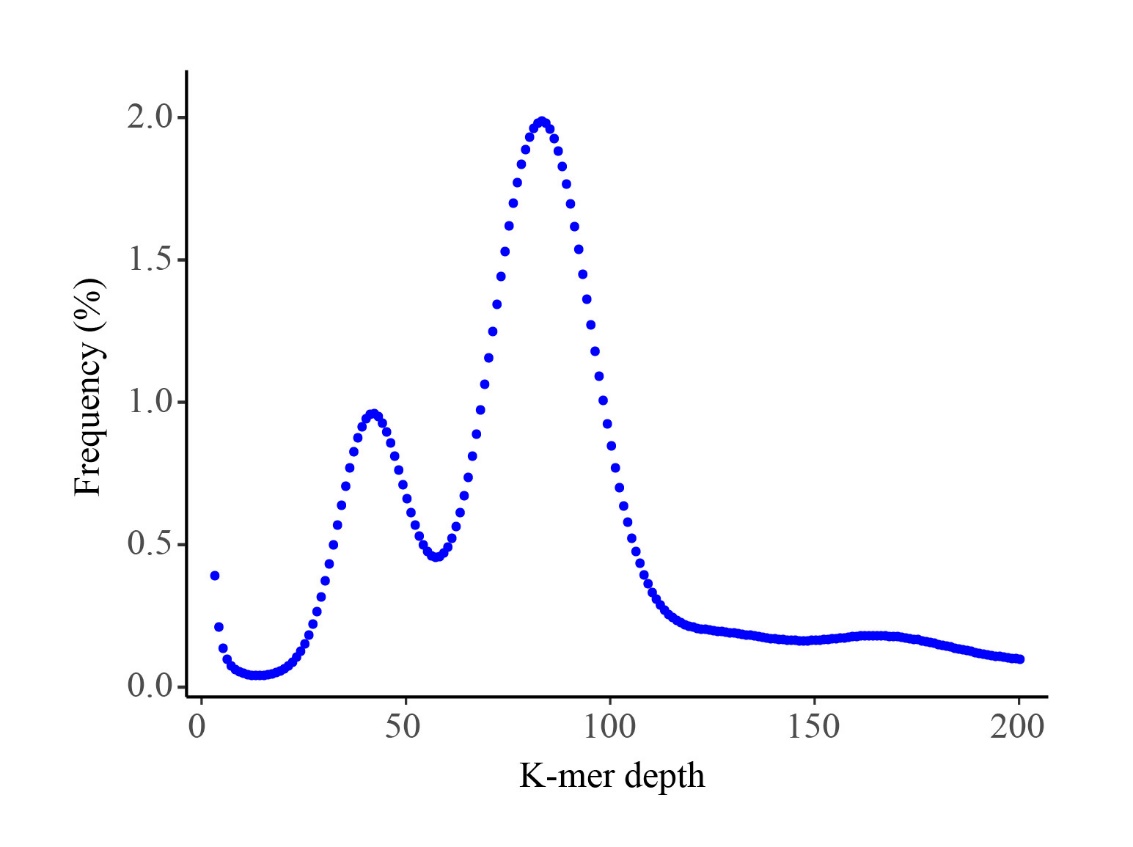


Supplementary figure1: Genome survey analysis of *C. heterophylla* based on k-mer = 19. The estimated genome size is 373.61 Mb. The repeat content and heterozygosity are 42.89% and 0.93%, respectively.


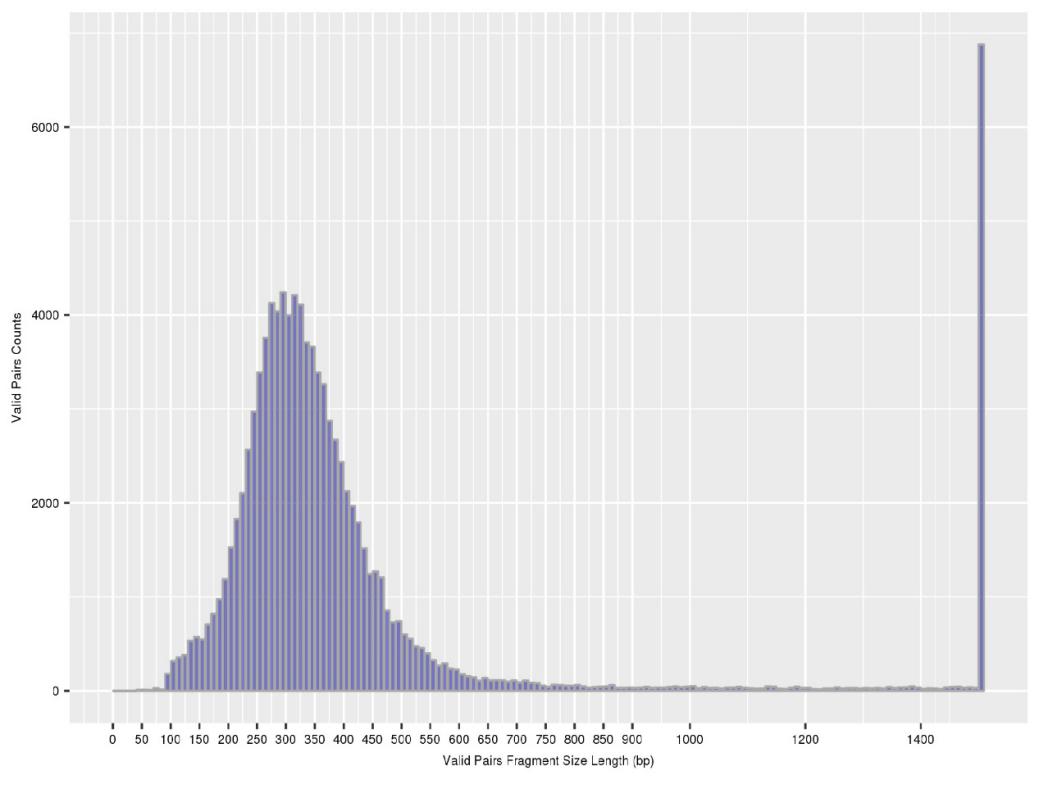


Supplementary figure2: Fragment size distribution of Hi-C read pairs.


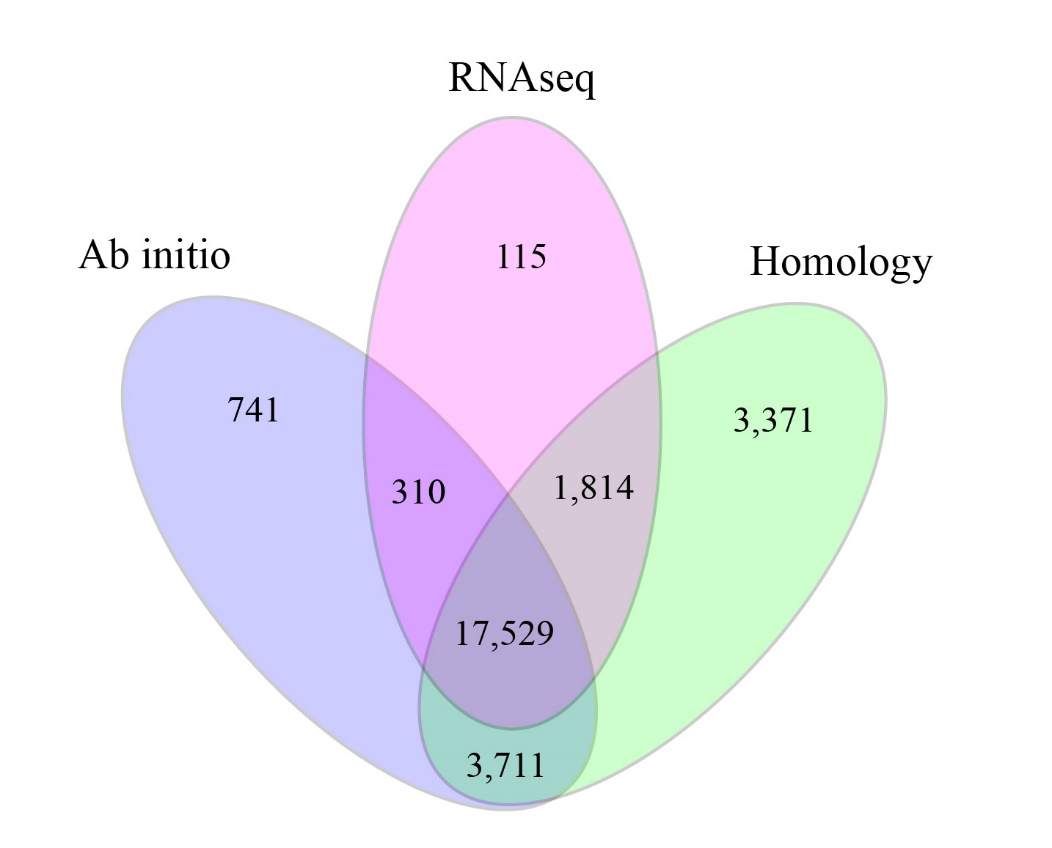


Supplementary figure3: Venn plot of predicted genes generated from ab initio, RNAseq and homology methods.
