# Supplementary material for: A chromosome-level reference genome of the hazelnut, Corylus heterophylla Fisch
Source: Gigascience. 2021 Apr 19;10(4):giab027. doi: 10.1093/gigascience/giab027 (PMC8054262; doi:10.1093/gigascience/giab027)
Supplement: giab027_Response_to_Reviewer_Comments_Original_Submission [file giab027_response_to_reviewer_comments_original_submission.pdf]

Author's Response To Reviewer Comments

Close

19 February, 2021
Editor : Dr. Hongling Zhou
GigaScience
Ms. No. GIGA-D-20-00312

Dear Dr. Hongling Zhou,
We appreciate the time and effort that you and reviewers dedicated to provide the helpful feedback on our manuscript "A chromosome-level reference genome of the hazelnut, Corylus heterophylla Fisch"(GIGA-D-20-00312). We have carefully revised our manuscript in light of your extensive and helpful comments and those of the reviewers. We have added the RRID of the biological tools obtained from SciCrunch.org database into the revised manuscript. In order to make the figure more neat and appropriate, we adjusted Figure 1 (C, D) and replaced them with new photos. We also revised the corresponding figure legends of Figure 1. Here we resubmit our revised manuscript to Journal of the GigaScience. Below please find a detailed response to the questions raised by the reviewers. During the proof reading and revision of this paper, Dr. Xin Chen has given us great help, so with the consent of all the authors, we hope to add him as one of the co-authors of this paper. Finally, the revised manuscript has obtained a language editing help from Charlesworth Author Services Team. We hope the revised manuscript would satisfy you and reviewers.

Thank you again for your time and effort.

Sincerely,

Lujun Wang

Response to Reviewer #1:
1 Reviewer's comment: Lines 46-50: A reference for these statements is needed.
Author's response: Thanks for the reviewer helpful comments for our work. As suggested, we added reference in line 52.

2 Reviewer's comment: Line 54: What is meant by "most economically wild"?
Author's response: Thanks to reviewer's attention. To avoid confusion, we rewrote this sentence as "Corylus heterophylla is one of the most important economic Corylus species. Among the 1.67 million ha of wild Corylus in China, C. heterophylla occupies 90% of the area." in the revised manuscript.

3 Reviewer's comment: Line-s 242-243: It appears that you are confusing ortholog groups with gene families. OrthoMCL is used to detect ortholog groups, not gene families.
Author's response: We agree with reviewer's opinion. We replaced the "gene families" to "ortholog groups" in the revised manuscript.

4 Reviewer's comment: Line 290: Hexadecyltrimethyl is missing the "l" at the end.
Author's response: Sorry for this spelling mistake. We have corrected this mistake in the revised manuscript.

Response to Reviewer #2:
1 Reviewer's comment: The accession IDs for NCBI and SRA were still missing from this version, I would like to request that the data be deposited to the repository upon publication of the assembly.
Author's response: Many thanks for reviewer's helpful suggestion. As suggested, we have add the NCBI accession IDs for genome (JADOBO000000000) and SRA (SRR12458330, SRR12458329, SRR12458328, SRR12458327) at Availability of supporting data section (lines 346-350)

2 Reviewer's comment: The paper needs proof reading and help from a native English speaking person.
Author's response: As reviewer's suggestion, the paper has been send to Charlesworth Author Services

Team for English language revision.

3 Reviewer’s comment: Line 16: economically and ecologically important nut tree
Author’s response: We have corrected this sentence in the revised manuscript.

4 Reviewer’s comment: Line 19: Nanopore (capital letter)
Author’s response: We have corrected the “nanopore” as “Nanopore” in the revised manuscript.

5 Reviewer’s comment: Line 29: bad english: molecular mechanism of hazelnut responsing to environmental stress and serve as a resource ->molecular mechanisms of how hazel nut responds to environmental stresses and serves as a resource (or similar)
Author’s response: As reviewer’s suggestion, we have corrected this sentence as “This genome resource will promote research on the molecular mechanisms of how the hazelnut responds to environmental stresses and serves as an important resource for genome-assisted improvement in cold and drought resistance of the Corylus genus” in the revised manuscript (lines 31-34).

6 Reviewer’s comment: 35: provides
Author’s response: As suggested, we have corrected the word “provide” as “provides” in the revised manuscript.

7 Reviewer’s comment: 36: There is a high content
Author’s response: We have corrected “are” as “is” in the revised manuscript.

8 Reviewer’s comment: 39 ranges (or varies between)
Author’s response: We revised this spelling mistake as “ranges” in the revised manuscript.

9 Reviewer’s comment: 45: Inadequate
Author’s response: We have revised the spelling mistake as “Inadequate” in the revise manuscript.

10 Reviewer’s comment: 54: "one of the most economically wild Corylus species" - what does this mean?
Author’s response: To avoid confusion, we have revised this sentence as “Corylus heterophylla is one of the most important economic Corylus species. Among the 1.67 million ha of wild Corylus in China, C. heterophylla occupies 90% of the area.” in the revised manuscript (lines 54-55).

11 Reviewer’s comment: 85 Qbit -> Qubit
Author’s response: We revised this spelling mistake as “Qubit” in the revised manuscript.

12 Reviewer’s comment: 109: fuorometry -> fluorometry
Author’s response: We revised the spelling mistake as “fluorometry” in the revised manuscript.

13 Reviewer’s comment: 253: this is the monocot - dicot split time
Author’s response: Thanks for reviewer’s comment. To avoid confusion, we revised this sentence as “The monocot-dicot split time (152 - 160 Mya) getting from TimeTree database was also used to calibrate the time estimation”.

Close
